# Supplementary material for: Mating can initiate stable RNA silencing that overcomes epigenetic recovery
Source: Nat Commun. 2021 Jul 9;12:4239. doi: 10.1038/s41467-021-24053-4 (PMC8270896; doi:10.1038/s41467-021-24053-4)
Supplement: Supplementary file 1 — Supplementary Information [file 41467_2021_24053_MOESM1_ESM.pdf]

Mating can initiate stable RNA silencing that overcomes epigenetic recovery

Sindhuja Devanapally*, Pravrutha Raman*, Mary Chey, Samual Allgood, Farida Ettefa, Maïgane Diop, Yixin Lin, Yongyi E Cho, and Antony M Jose
*equal contribution

This supplement contains the following text, figures and tables.

Supplementary Discussion

Supplementary Figures
Supplementary Fig. 1. Mating-induced silencing can be robustly initiated despite modifications of the gene sequence.
Supplementary Fig. 2. Mating-induced silencing does not occur with other germline genes and is not explained by changes in transcript abundance or localization in the germline.
Supplementary Fig. 3. Schematics of key transgenes, schematic of the piRNA-mediated silencing pathway, and tests of its requirement for two forms of RNA silencing.
Supplementary Fig. 4. Mating-induced silencing results in quantitative reduction of *mCherry* and *gfp* transcripts and protein within the germline in cross progeny.
Supplementary Fig. 5. Protection and maintenance of mating-induced silencing.
Supplementary Fig. 6. Mating can trigger maintenance of transgenerational silencing by quantitative reduction of transcripts without detectable changes in H3K9me across generations.
Supplementary Fig. 7. Variants of *T* can recover from silencing by a heritable silencing signal acting in *trans*.
Supplementary Fig. 8. The same target sequence can show variability in transgenerational silencing within the germline upon feeding RNAi.

Supplementary Tables
Supplementary Table 1. Reports on heritability of RNA silencing suggest variable stability of induced RNA silencing.
Supplementary Table 2. Comparison of mating-induced silencing with related epigenetic phenomena.
Supplementary Table 3. Strains used.
Supplementary Table 4. Oligonucleotides used.
Supplementary Table 5. Reagents used for Cas9-mediated genome editing.

Supplementary References

Supplementary Discussion

Maintenance of silencing initiated by mating occurs through mechanisms that are reminiscent of paramutation/RNAe

Paramutation refers to meiotically heritable changes in gene expression transferred from one allele ("paramutagenic") to another allele ("paramutable") when they interact within a cell (reviewed in ref. 1). In addition to similar heritability, both paramutation[2-6] and mating-induced silencing rely on small RNAs to spread silencing from one locus to another homologous locus. However, there are some aspects of paramutation that were found to be different from mating-induced silencing when tested. First, a paramutagenic allele often requires associated repetitive sequences[7-9]. Second, how a paramutagenic allele first arises remains unclear[1]. Third, while some alleles are paramutable, others are not, for reasons that are unknown[2]. The reliability of initiating and also protecting from meiotically heritable silencing at a defined single-copy locus described in this study will be useful in discovering possible shared mechanisms that have remained unclear in the ~65 years since the original discovery of paramutation in maize[3].

The unpredictable silencing that occurs at some single-copy reporter transgenes within the *C. elegans* germline has been called RNA-induced epigenetic silencing or RNAe[10-15]. Some studies of RNAe[13,14,16], but not others (p.94 in ref. 12), report a requirement for PRG-1 only in the initiation of silencing during RNAe, which is similar to the requirement for maternal PRG-1 in mating-induced silencing (Fig. 2a,b). However, while *hrde-1/wago-9* was uniformly reported to be required for the maintenance of silencing, our results reveal a requirement for HRDE-1 in both the initiation and the maintenance of mating-induced silencing (Fig. 2a, Fig. 3c). Transgenes silenced through RNAe are associated with specific genome sequences or a differential subset of small RNAs compared to unsilenced transgenes[16-18] but it remains unclear whether these associated properties of the silenced genes are the cause or consequence of silencing. Nevertheless, a model proposing RNAe as a response to foreign or non-self DNA emerged[10-14]. This model is inadequate because the same target sequence (e.g. *mCherry*, *gfp*) can be either silenced or expressed within the germline (Figs. 1a, 6a and S2a; refs. 13,14,16,17) and endogenous genes are subjected to transgenerational silencing through similar PRG-1- and HRDE-1-dependent mechanisms[19-24]. Furthermore, the features of a transgene that trigger silencing are unknown. Tethering the Argonaute CSR-1 to the nascent transcript[11] or adding intronic sequences that are found in native germline-expressed genes[25] can increase the frequency of expression of a foreign sequence but does not itself determine whether a sequence is expressed. Thus, despite these efforts, the factors that enable stable expression or silencing of a gene across generations remain unclear.

Unlike RNAe, mating-induced silencing can be predictably initiated at the population level with stochastic differences in initiation at the individual level, which together provide a reliable assay for evaluating how organisms establish stable expression or silencing of a gene. Our analyses suggest that the decision to express paternal foreign sequences (*mCherry* and *gfp*) is re-evaluated in each generation based upon maternal mRNA (Fig. 2). Although mating-induced silencing is not a general property of genes (Supplementary Fig. 2a, b), a similar silencing phenomenon with dependence on maternal mRNA has been observed for the endogenous gene *fem-1*[26]. However, it is unknown whether *fem-1* silencing also shares the *trans* silencing properties and genetic requirements of mating-induced silencing.

Taken together, the mating-induced silencing documented in this study provides a reliable model for analyzing epigenetic mechanisms that dictate expression or silencing of a sequence in every generation in otherwise wild-type animals.

Supplementary Figures and Figure Legends

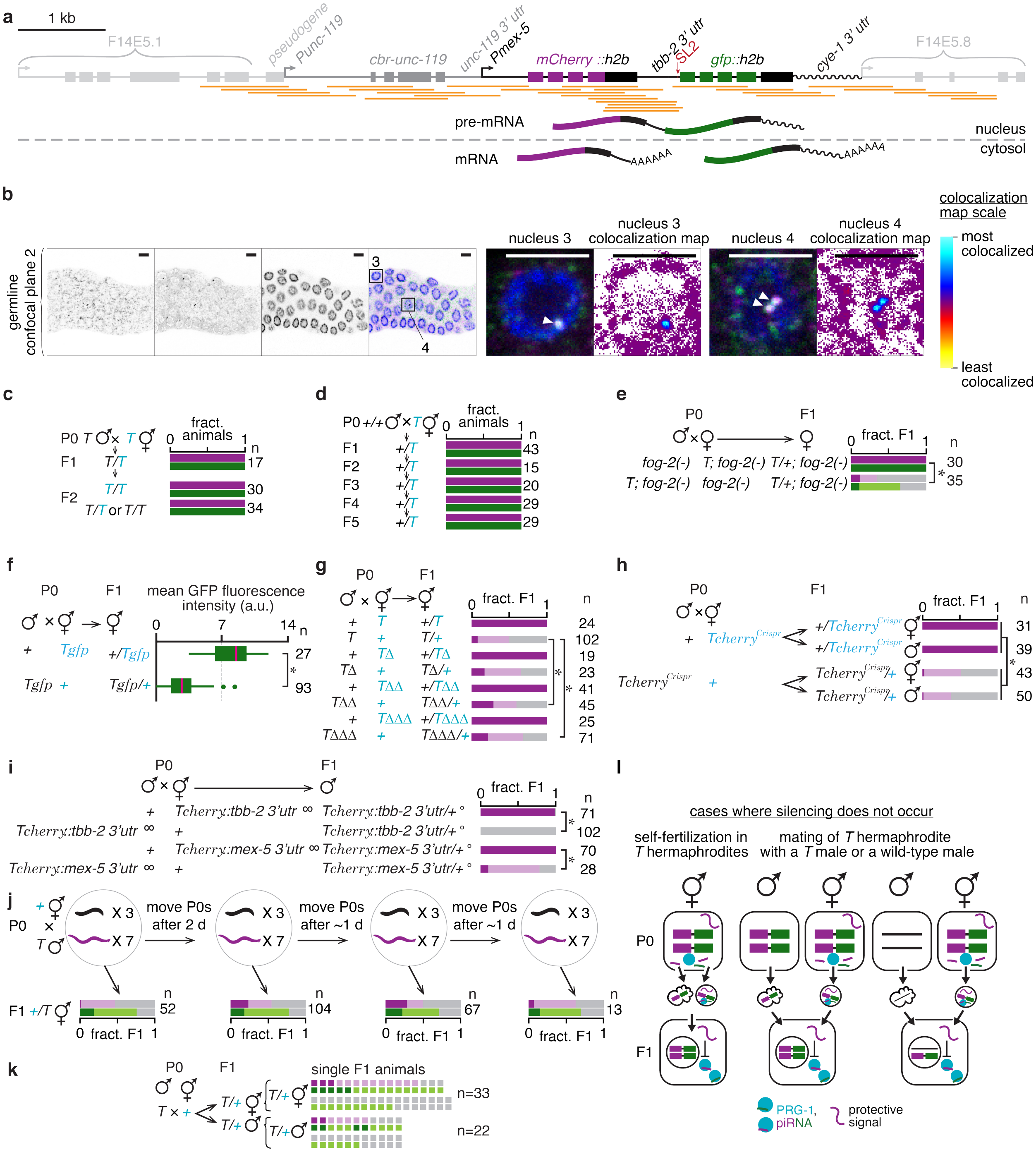

Supplementary Fig. 1. Mating-induced silencing can be robustly initiated despite modifications of the gene sequence.

a Schematic of *T* (*oxSi487*: *Pmex-5::mCherry::h2b::tbb-2 3' utr::gpd-2 operon::gfp::h2b::cye-1 3' utr*) within its genomic context where it is present as a single copy transgene as verified by PCR and Sanger sequencing. The transgene consists of *mCherry* and *gfp* genes tagged to *histone 2b* (*his-58* or *his-66*) arranged in an operon, and is presumably transcribed into one nascent transcript (pre-mRNA) with both *mCherry::h2b* and *gfp::h2b* present as two separate mature

transcripts (mRNA) in the cytosol. Orange lines correspond to sequence stretches verified by individual Sanger sequencing experiments. The genes surrounding the insertion site of *T* on chromosome II are shown.

b smFISH against *mCherry* and *gfp* in dissected gonads of animals expressing *T* reveals that *mCherry* RNA and *gfp* RNA colocalize as one or two spots (white arrowheads) within nuclei. A different confocal plane imaged from the same dissected gonad in Fig. 1b reveals different nuclei with colocalized *mCherry* and *gfp*. Colocalization heat map represents the extent of overlap between smFISH pixels corresponding to *mCherry* RNA and *gfp* RNA as indicated by the scale. *mCherry* RNA image was obtained by subtracting DAPI signal from images that contain combined pixels corresponding to mCherry::H2B protein (with which DAPI signal completely overlaps) and *mCherry* RNA fluorescence.

c Males and hermaphrodites expressing *T* were mated, and fluorescence was scored in cross progeny (F1) and self-fertilized grand-progeny (F2) that inherited only the grand-maternal allele or only the grand-paternal allele or both. F1 data shown here is the same as that in Fig. 1d.

d Wild-type males were mated with *T* hermaphrodites and hemizygous cross progeny (F1) as well as descendant hemizygous self-progeny (F2 through F5) were scored. In contrast to previous reports[27], we find that *T* is not subject to meiotic silencing by unpaired DNA[28].

e Mutation of *fog-2* feminizes the germline in 100% of hermaphrodites but has no effect in males[29]. Feminized mothers expressing *T* were used in a control cross and those without *T* were used in a cross to initiate mating-induced silencing.

f Germline GFP fluorescence from hemizygous *Tgfp*/+ cross progeny shown in Fig. 1e was quantified. Box plot characteristics are: red line, median; box limits, upper and lower quartiles; whiskers, 1.5x interquartile range; points, outliers.

g Animals expressing variants of *T* were mated with non-transgenic animals and cross progeny were scored.

h Animals expressing *Tcherry* inserted into the genome using CRISPR-Cas9 were mated with non-transgenic animals and cross progeny were scored.

i Animals expressing *Tcherry* with altered 3' UTR were mated to non-transgenic animals and cross progeny were scored. To reverse spontaneous transgene silencing[16,25] upon genome insertion, *hrde-1(-)* was introduced (∞) into P0 transgenic animals resulting in heterozygous *hrde-1(+/-)* cross progeny (°).

j *T* males and non-transgenic hermaphrodites were mated and cross progeny that were laid in the first 48 hours (2 days, 2 d) or in subsequent ~24 hour intervals (1 day, 1 d), were collected after moving the P0s to fresh plates. While silencing triggered by parental ingestion of dsRNA is less effective in later progeny[30,31], silencing triggered by mating can be equally effective in early and in late progeny.

k Cross progeny males and hermaphrodites that inherited *T* paternally were scored. Scoring data from the crosses in Fig. 1d is depicted as a coloured pair of boxes to show mCherry and GFP fluorescence in each individual.

l Schematics that depict possible explanations for lack of silencing: *left*, when homozygous *T* hermaphrodites self-fertilize using transgenic sperm carrying *T* and oocytes that express *T* and carry PRG-1-dependent piRNAs complementary to *T*, self-progeny remain unsilenced possibly due to a protective signal derived from *T* transmitted from the hermaphrodite parent through the oocyte into progeny; *middle*, when *T* males are mated with hermaphrodites expressing *T*, cross progeny remain unsilenced due to the protective signal transmitted from the hermaphrodite parent through the oocyte into progeny; *right*, when wild-type males are mated with

hermaphrodites expressing *T*, cross progeny inherit the protective signal through the oocyte that prevents initiation of mating-induced silencing.

Chromosomes with a *dpy* marker (blue font) and number of animals scored (n) are indicated. Scoring of silencing is as in Fig. 1. Asterisks indicate $P < 0.05$ using χ^2 test (e, g, h, i) or two-sided Student's t-test (f). Scale bars, 5 µm (b). Also see 'Genetic Crosses' under Supplementary Methods.

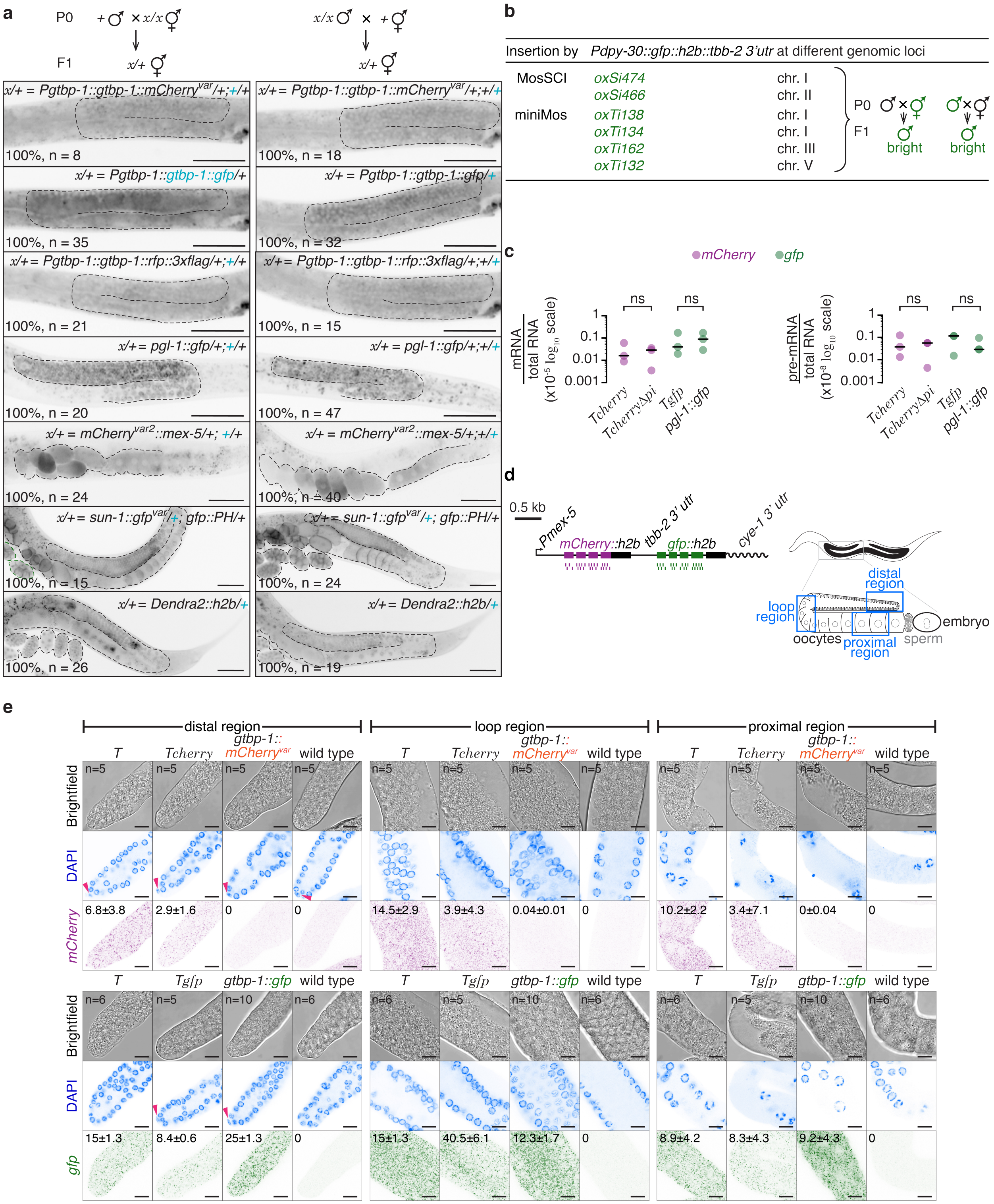

Supplementary Fig. 2. Mating-induced silencing does not occur with other germline genes and is not explained by changes in transcript abundance or localization in the germline.
a and b Endogenous genes tagged with reporter sequences (*gtbp-1::mCherry*^{var}, *gtbp-1::gfp*, *gtbp-1::rfp::3xflag*, *mCherry*^{var2}*::mex-5* and *pgl-1::gfp*) using CRISPR-Cas9-mediated genome

editing and transgenes made using miniMos[14] (*Pdpy-30::gfp::h2b::tbb-2 3' utr*), MosSCI (*Pdpy-30::gfp::h2b::tbb-2 3' utr*, *sun-1::gfp*^{var} and *Pmex-5::Dendra2::h2b::tbb-2 3' utr*), or bombardment (*Ppie-1::gfp::PH(PLCdelta1)*) were tested for susceptibility to mating-induced silencing. Germlines of representative cross progeny at L4 or adult stage are outlined and percentages of animals showing the expression depicted in the image are indicated in **a**.
c *mCherry* and *gfp* mRNA levels were measured by qRT-PCR between animals expressing *Tcherry* or *TcherryΔpi* and *Tgfp* or *pgl-1::gfp* respectively. The fold change of pre-mRNA:mRNA ratios between *Tcherry* and *TcherryΔpi* is ~0.5 and between *Tgfp* and *pgl-1::gfp* is ~2.2. ns indicate $P > 0.05$ using two-sided Student's t-test.
d smFISH probes that hybridize to *mCherry* or *gfp* exonic RNA (*left*) and schematics of germline regions imaged for smFISH or protein fluorescence using confocal microscopy (this figure, Fig. 1, Supplementary Fig. 1, Supplementary Fig. 4, and Supplementary Fig. 6) are indicated (*right*).
e Animals that express sequence variants of *mCherry* (*top*) or *gfp* (*bottom*) genes were subjected to smFISH against *mCherry* or *gfp* transcripts within dissected gonads. Genotypes of *gtbp-1::gfp* and *gtbp-1::mCherry*^{var} animals shown here are the same as those shown in **a**. Numbers within fluorescence images refer to number of RNAs per 100 μm^2 with standard error of the mean. Animals with median values of fluorescence or RNA signal in the distal region are represented along with the loop and proximal regions within the same animals. Pink arrowheads indicate the nucleus of the distal tip cell, when included in the imaging plane.
Chromosomes with a recessive marker (blue font) and numbers of animals scored or imaged (n) are indicated. Scale bars are 50 µm (a) or 10 µm (e).

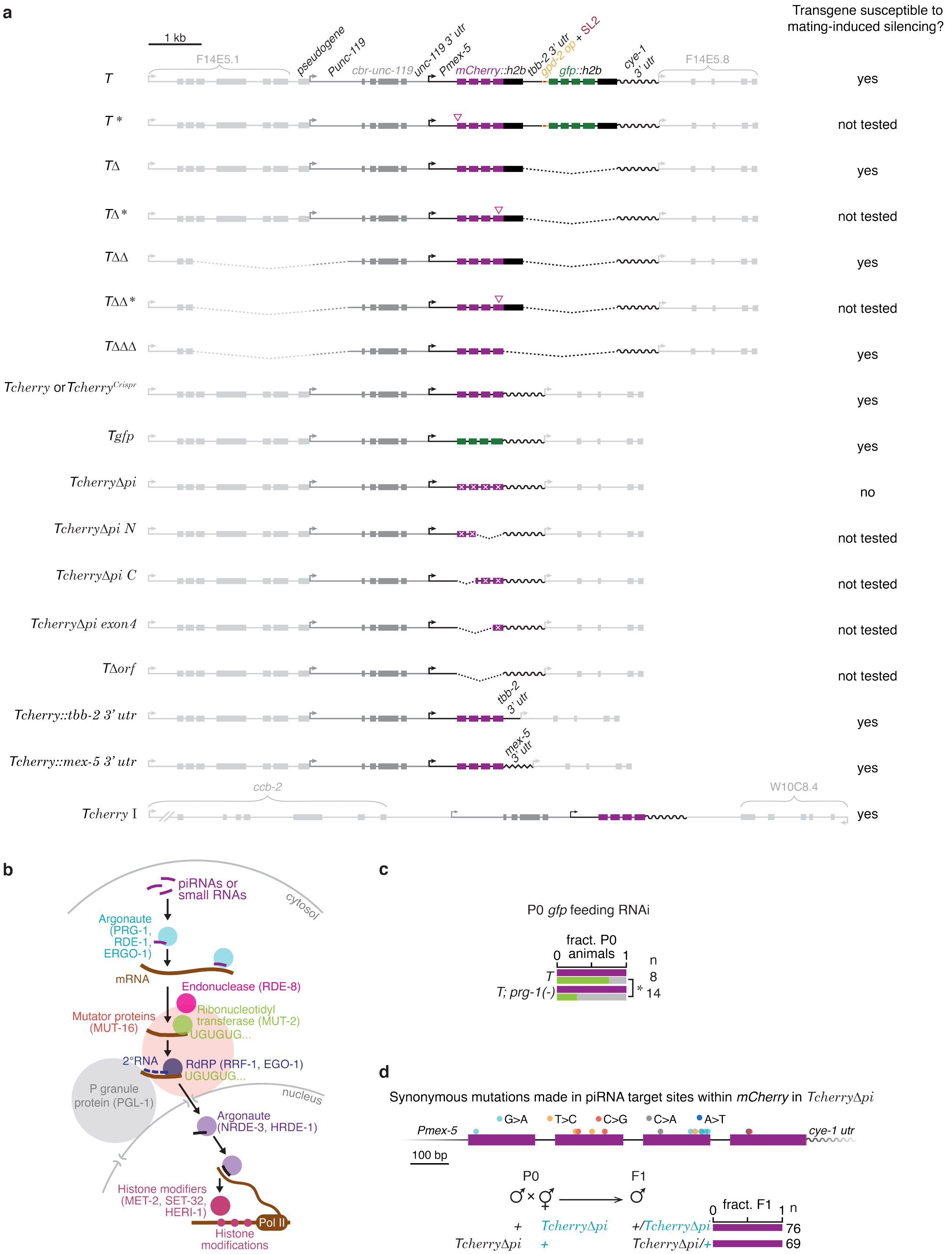

Supplementary Fig. 3. Schematics of key transgenes, schematic of the piRNA-mediated silencing pathway, and tests of its requirement for two forms of RNA silencing.

a Schematics of *T*, of serial deletions and/or indels of *T* and of minimal variants of *T* that were newly integrated into a naive genome. Successive deletions that remove *gfp* and *tbb-2 3' utr* (*T*Δ), a ~3 kb region upstream of the *unc-119(+)* coding region (*T*ΔΔ), and *h2b* (*T*ΔΔΔ) are depicted in their genomic context, along with variations that in addition contain small indels (*T**, *T*Δ*, *T*ΔΔ*). *Tcherry*, *Tcherry*^{Crispr}, *Tgfp*, *Tcherry*Δ*pi*, *Tcherry::tbb-2 3' utr*, *Tcherry::mex-5 3' utr* and *Tcherry* on chromosome I were integrated independently of each other. *Tcherry*Δ*pi N*, *Tcherry*Δ*pi C* and *Tcherry*Δ*pi exon4* were generated by independently editing animals carrying *Tcherry*Δ*pi*. *T*Δ*orf* was made by deleting *mCherry* in animals carrying *Tcherry*. The susceptibility of each variant to mating-induced silencing is indicated on the right. Certain variants were not tested for mating-induced silencing because they either contain a premature stop codon or do not contain a coding region. Also see Supplementary Table 5.
b Working model of RNA silencing mechanisms in *C. elegans* based on prior studies. Schematic depicting the described role of different components of the small RNA pathway that were examined for their requirement in initiation (Fig. 2) or maintenance (Fig. 3c) of mating-induced silencing[32-34]. Within the germline, secondary (2°) RNA production is not always correlated with gene silencing[32,35,36].
c Animals expressing *T* in a wild-type or *prg-1(-)* background were exposed to *gfp* RNAi or control RNAi for one generation (P0 RNAi) and scored.
d Schematic of synonymous changes in predicted piRNA sites within *mCherry* is depicted. Animals expressing *Tcherry* lacking piRNA binding sites (*Tcherry*Δ*pi*) were mated with non-transgenic animals, and cross progeny males were scored. Scoring of silencing (c and d) is as in Fig. 1c. Chromosomes with a *dpy* marker (blue font in d) and number of animals scored (n) are indicated. Asterisks indicate $P < 0.05$ using χ^2 test.

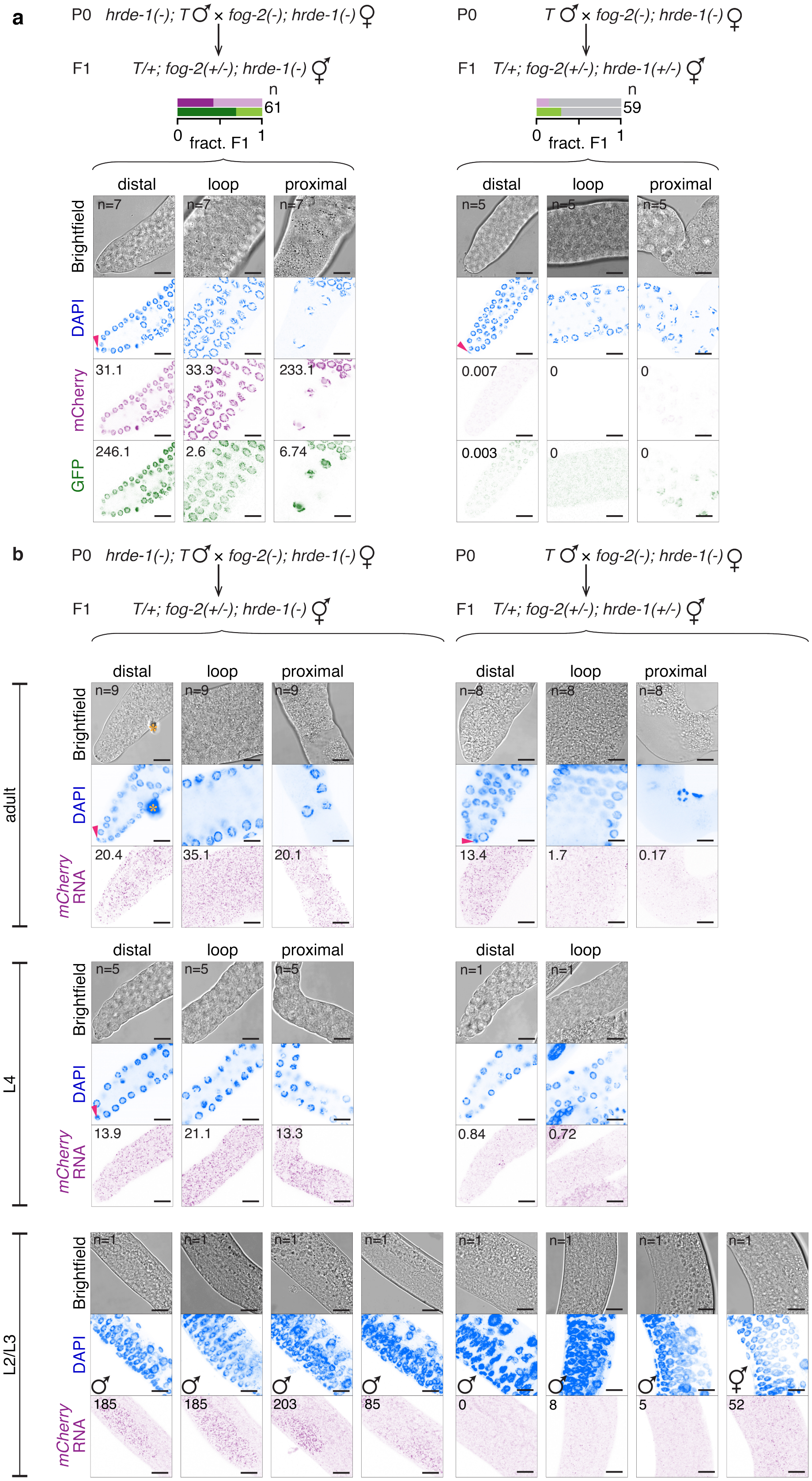
a
P0 hrde-1(-); T ♂ × fog-2(-); hrde-1(-) ♀
F1 T/+; fog-2(+/-); hrde-1(-) ⚥
n
61
0 fract. F1 1
P0 T ♂ × fog-2(-); hrde-1(-) ♀
F1 T/+; fog-2(+/-); hrde-1(+/-) ⚥
n
59
0 fract. F1 1
distal
loop
proximal
Brightfield
DAPI
mCherry
GFP
n=7
n=5
31.1
33.3
233.1
246.1
2.6
6.74
0.007
0
0.003
b
P0 hrde-1(-); T ♂ × fog-2(-); hrde-1(-) ♀
F1 T/+; fog-2(+/-); hrde-1(-) ⚥
P0 T ♂ × fog-2(-); hrde-1(-) ♀
F1 T/+; fog-2(+/-); hrde-1(+/-) ⚥
adult
mCherry RNA
n=9
n=8
20.4
35.1
20.1
13.4
1.7
0.17
L4
n=5
n=1
13.9
21.1
13.3
0.84
0.72
L2/L3
185
185
203
85
0
8
5
52

Supplementary Fig. 4. Mating-induced silencing results in quantitative reduction of *mCherry* and *gfp* transcripts and protein within the germline in cross progeny.

Initiation of mating-induced silencing was measured by protein fluorescence microscopy or smFISH against *mCherry* RNA in dissected gonads of cross progeny that were impaired for (*top*) or susceptible to (*bottom*) mating-induced silencing. While control cross progeny would ideally be derived from *T* hermaphrodites mated with wild-type males, we did not examine progeny from such a cross because maternally deposited transcripts from *T* would add to progeny transcripts detected. Instead, mating-induced silencing was attempted in the parental and zygotic absence of *hrde-1*, which is required for initiation of silencing (refer to Fig. 2a, b), thus allowing us to examine transcripts from *T* in the absence of silencing in cross progeny.

a *T* or *T; hrde-1(-)* males were mated with *hrde-1(-); fog-2(-)* females and fluorescence due to mCherry::H2B and GFP::H2B in cross progeny was scored by eye after imaging (*top*) or by quantifying confocal slices of indicated regions of dissected gonads (*bottom*). Scoring of silencing by eye and number of animals assayed are as in Fig. 1c. For confocal images of cross progeny in left panel (control cross), fluorescence intensity values (arbitrary units) ranged from 5.3 to 42.6 (mCherry, distal), 0.0006.4 to 246.1 (GFP, distal), 4.6 to 39.1 (mCherry, loop), 0.2 to 14.2 (GFP, loop), 0 to 250.5 with 5/7 animals >5.0 (mCherry, proximal), 0.002 to 7.9 with 4/7 animals ≥2.2 (GFP, proximal). For confocal images of cross progeny in right panel (mating-induced silencing cross), fluorescence intensity values (arbitrary units) ranged from 0.0005 to 24.5 (mCherry, distal), 0.001 to 0.6 (GFP, distal), 0 to 254 with 3/5 animals = 0 (mCherry, loop), 0.0007 to 254.8 with 4/5 animals ≤0.8 (GFP, loop), 0 to 6.5 with 4/5 animals = 0 (mCherry, proximal), 0.0007 to 2.9 with 4/5 animals ≤0.03 (GFP, proximal).

b smFISH of *mCherry* RNA in cross progeny adults obtained from a mating as in panel a. For confocal images of cross progeny in left panel (control cross), *mCherry* RNAs per 100 μm^2 ranged from 2.5 to 33.8 (adult, distal), 1.5 to 42.6 (adult, loop), 0.6 to 37 with 8/9 animals >15.8 (adult, proximal), 0.8 to 18.9 (L4, distal), 17.3 to 29.2 (L4, loop), 0.12 to 15.8 with 4/5 animals >6.9 (L4, proximal). For confocal images of cross progeny in right panel (mating-induced silencing cross), *mCherry* RNAs per 100 μm^2 ranged from 0.9 to 27.8 (adult, distal), 0 to 19 (adult, loop), 0 to 26.2 with 7/8 animals <9.6 (adult, proximal).

Pink arrowhead, nucleus of the distal tip cell and orange asterisk, non-specific signal. Numbers within fluorescence images refer to mean fluorescence intensity per unit area measured in arbitrary units (a) or number of RNAs per 100 μm^2 (b). Animals with median values of protein or RNA fluorescence signal in the distal region are represented along with the loop and proximal regions within the same animals. Scoring of silencing (a, *top*) is as in Fig. 1. Scale bars are 10 µm. Number (n) of animals scored (a) or imaged per region using confocal microscopy (a and b) is indicated.

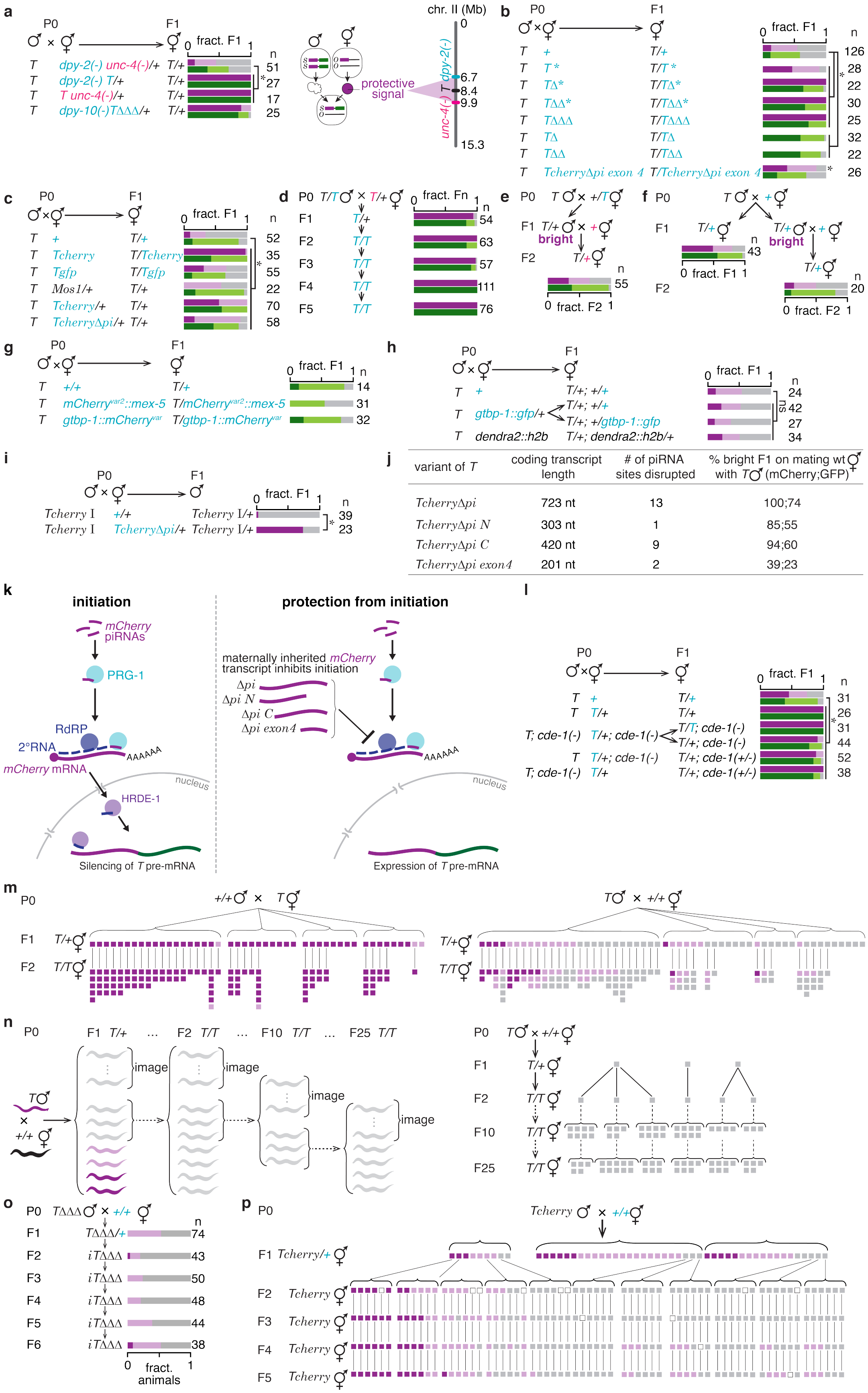
a
P0
F1
fract. F1
n
T dpy-2(-) unc-4(-)/+ T/+ 51
T dpy-2(-) T/+ T/+ 27
T T unc-4(-)/+ T/+ 17
T dpy-10(-)TΔΔΔ/+ T/+ 25
protective signal
chr. II (Mb)
dpy-2(-)
unc-4(-)
6.7
8.4
9.9
15.3
b
T + T/+ 126
T T* T/T* 28
T TΔ* T/TΔ* 22
T TΔΔ* T/TΔΔ* 30
T TΔΔΔ T/TΔΔΔ 25
T TΔ T/TΔ 32
T TΔΔ T/TΔΔ 22
T TcherryΔpi exon 4 T/TcherryΔpi exon 4 26
c
T + T/+ 52
T Tcherry T/Tcherry 35
T Tgfp T/Tgfp 55
T Mos1/+ T/+ 22
T Tcherry/+ T/+ 70
T TcherryΔpi/+ T/+ 58
d
P0 T/T × T/+
fract. Fn
F1 T/+ 54
F2 T/T 63
F3 T/T 57
F4 T/T 111
F5 T/T 76
e
P0 T × +/T
F1 T/+ × +
bright
F2 T/+
fract. F2
55
f
P0 T × +
F1 T/+ T/+ × +
bright
43
fract. F1
F2 T/+
fract. F2
20
g
T +/+ T/+ 14
T mCherryvar2::mex-5 T/mCherryvar2::mex-5 31
T gtbp-1::mCherryvar T/gtbp-1::mCherryvar 32
h
T + T/+; +/+ 24
T gtbp-1::gfp/+ T/+; +/+ 42
T/+; +/gtbp-1::gfp 27
T dendra2::h2b T/+; dendra2::h2b/+ 34
ns
i
Tcherry I +/+ Tcherry I/+ 39
Tcherry I TcherryΔpi/+ Tcherry I/+ 23
j
variant of T | coding transcript length | # of piRNA sites disrupted | % bright F1 on mating wt with T (mCherry;GFP)
TcherryΔpi | 723 nt | 13 | 100;74
TcherryΔpi N | 303 nt | 1 | 85;55
TcherryΔpi C | 420 nt | 9 | 94;60
TcherryΔpi exon4 | 201 nt | 2 | 39;23
k
initiation
mCherry piRNAs
PRG-1
RdRP
2°RNA
AAAAAA
mCherry mRNA
nucleus
HRDE-1
Silencing of T pre-mRNA
protection from initiation
maternally inherited mCherry transcript inhibits initiation
Δpi
Δpi N
Δpi C
Δpi exon4
Expression of T pre-mRNA
l
T + T/+ 31
T T/+ T/+ 26
T; cde-1(-) T/+; cde-1(-) T/T; cde-1(-) 31
T/+; cde-1(-) 44
T T/+; cde-1(-) T/+; cde-1(+/-) 52
T; cde-1(-) T/+ T/+; cde-1(+/-) 38
m
P0 +/+ × T
F1 T/+
F2 T/T
T × +/+
n
P0 F1 T/+ ... F2 T/T ... F10 T/T ... F25 T/T
T × +/+
image
P0 T × +/+
F1 T/+
F2 T/T
F10 T/T
F25 T/T
o
P0 TΔΔΔ × +/+
F1 TΔΔΔ/+ 74
F2 iTΔΔΔ 43
F3 iTΔΔΔ 50
F4 iTΔΔΔ 48
F5 iTΔΔΔ 44
F6 iTΔΔΔ 38
fract. animals
p
P0 Tcherry × +/+
F1 Tcherry/+
F2 Tcherry
F3 Tcherry
F4 Tcherry
F5 Tcherry

Supplementary Fig. 5. Protection and maintenance of mating-induced silencing.
a *T* males were mated with genetically marked hermaphrodites and animals with paternally inherited *T* were scored. Schematic: maternal presence of *T*ΔΔΔ protects paternally inherited *T* from mating-induced silencing, suggesting that the oocyte carries a separable protective signal derived from a ~3.2 Mb region between *dpy-2* and *unc-4*, which are linked to *T*.
b and c *T* males were mated with non-transgenic hermaphrodites or hermaphrodites expressing a variant of *T* or *Mos1* transposon in the same genomic position as *T* and paternally inherited *T* was scored in resulting cross progeny. Control cross progeny to compare with *T/Tcherry*Δ*pi exon 4* cross progeny in this panel are shown in Fig. 2e.
d Hemizygous *T/+* hermaphrodites were mated with homozygous *T* males containing a recessive marker and hemizygous cross progeny (F1) as well as four generations of homozygous descendants (F2 through F5) were scored.
e and f Male progeny with bright mCherry fluorescence that were protected from initiation (e) or that escaped initiation of mating-induced silencing (f) were tested for mating-induced silencing.
g and h Males expressing *T* were mated with hermaphrodites expressing genes with homologous protein (g) or DNA (h) sequences, and fluorescence of GFP (g) or mCherry (h) from paternally inherited *T* was scored in cross progeny.
i Mating-induced silencing of *Tcherry* expressed from chromosome I could be protected by maternally expressed *Tcherry*Δ*pi* on chromosome II.
j Table indicating the length of coding regions and number of disrupted piRNA target sites[37] in *Tcherry*Δ*pi* or its derivatives, along with the percentage of bright cross progeny obtained upon mating hermaphrodites expressing one of these variants with *T* males. The percentages indicated here represent data from Fig. 2e and panel b in this figure.
k Model depicting one possible explanation for how protection from mating-induced silencing occurs in cross progeny. During initiation of mating-induced silencing, maternally inherited PRG-1 stabilizes complementary *mCherry* piRNAs which bind the *mCherry* mRNA made in progeny, resulting in recruitment of an RdRP to produce secondary small RNAs, which are then used by HRDE-1 to cause silencing of the pre-mRNA transcript comprising both *mCherry* and *gfp* in the nucleus (*left*). In protection from mating-induced silencing, it is possible that maternally inherited fragments of *mCherry* act as a sponge to soak up secondary RNAs, thus preventing the silencing of the pre-mRNA containing both *mCherry* and *gfp* transcript in the nucleus (*right*). Consistent with this model, the level of protection (i.e. the percentage of animals with bright mCherry and GFP fluorescence) is directly proportional to the length of the maternal transcript (see j). In other words, the longer the maternally inherited transcript, the more the capacity for it to soak up secondary small RNAs in progeny. See text for additional possibilities.
l Mutants of a CSR-1 pathway gene, *cde-1,* were used to test parental and zygotic requirement for protection.
m Animals expressing *T* were mated with wild-type animals in four independent crosses (brackets) and mCherry fluorescence was scored in hemizygous cross progeny and in homozygous grand progeny. Each box indicates fluorescence intensity of a single adult animal and lines indicate descent. Once initiated, mating-induced silencing persists despite passage of *T* through oocytes of hermaphrodites and is therefore unlike genomic imprinting[38,39], where passage of *T* through oocytes is expected to revive expression.
n F2 ‘off’ progeny (from **m**) obtained after initiation of mating-induced silencing were propagated without further selection by self-fertilization for 23 generations as indicated by the passaging scheme. mCherry fluorescence intensity was measured in animals (boxes) at F1, F2,

F10 and F25 generations from three independent P0 crosses. At each generation indicated, siblings of the animals that were passaged were scored. Presence of the transgene was verified by genotyping in F1 and F2 generations.

o *T*ΔΔΔ males were mated with non-transgenic hermaphrodites and scoring was done in cross progeny (F1) and in descendants propagated blindly from 'off' F1 animals.

p *Tcherry* males were mated with non-transgenic hermaphrodites in three independent crosses and cross progeny belonging to each fluorescence level were singled out to give F2 animals. From F2 through F5, a single animal was blindly passaged and a single descendant was scored. Empty box indicates that the animal could not be scored because it was lost after being passaged on to a fresh plate, but only after having laid eggs, which enabled the continued scoring of its descendants.

Scoring of silencing is as in Fig. 1c. Chromosomes with a recessive marker (blue font in **o** and **p**) and numbers of animals scored (n) are indicated. Asterisks indicate $P < 0.05$ using χ^2 test.

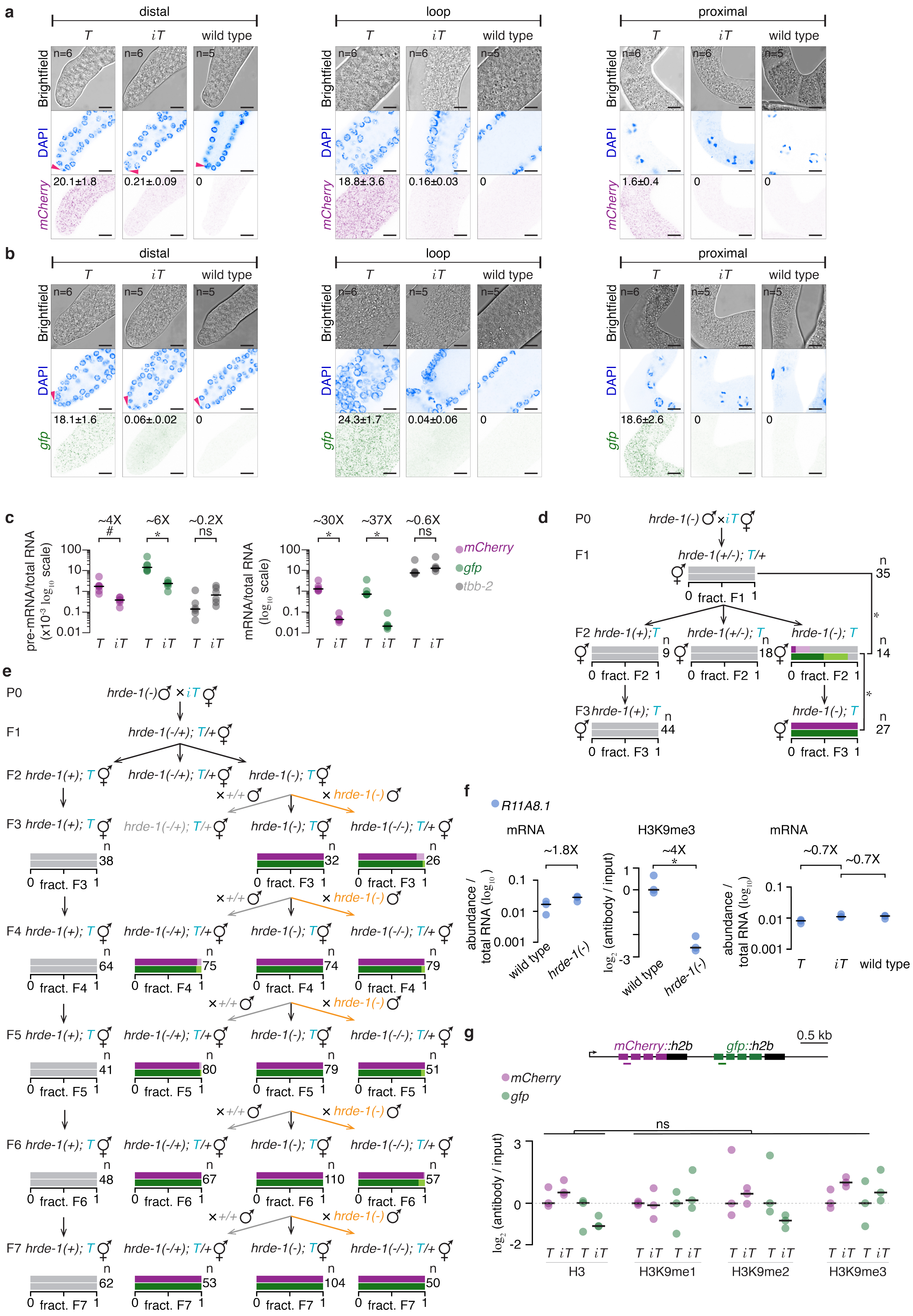
a
distal
loop
proximal
T
iT
wild type
Brightfield
DAPI
mCherry
n=6
n=5
20.1±1.8
0.21±0.09
0
18.8±3.6
0.16±0.03
1.6±0.4
b
gfp
18.1±1.6
0.06±0.02
24.3±1.7
0.04±0.06
18.6±2.6
c
~4X
~6X
~0.2X
~30X
~37X
~0.6X
#
*
ns
pre-mRNA/total RNA (x10^-3 log10 scale)
mRNA/total RNA (log10 scale)
mCherry
gfp
tbb-2
d
P0
F1
F2
F3
hrde-1(-) × iT
hrde-1(+/-); T/+
hrde-1(+); T
hrde-1(+/-); T
hrde-1(-); T
fract. F1
fract. F2
fract. F3
n
35
9
18
14
44
27
e
hrde-1(-) × iT
hrde-1(-/+); T/+
hrde-1(+); T
hrde-1(-); T
×+/+
×hrde-1(-)
hrde-1(-/+); T/+
hrde-1(-/-); T/+
F4
F5
F6
F7
38
32
26
64
75
74
79
41
80
79
51
48
67
110
57
62
53
104
50
fract. F4
fract. F5
fract. F6
fract. F7
f
R11A8.1
mRNA
H3K9me3
~1.8X
~4X
~0.7X
abundance / total RNA (log10)
log2 (antibody / input)
wild type
hrde-1(-)
wild type
g
mCherry::h2b
gfp::h2b
0.5 kb
H3
H3K9me1
H3K9me2
H3K9me3

Supplementary Fig. 6. Mating can trigger maintenance of transgenerational silencing by quantitative reduction of transcripts without detectable changes in H3K9me across generations.

a and b smFISH of *mCherry* (**a**) or *gfp* (**b**) exonic RNA was performed in indicated regions of dissected gonads of adult *T*, *iT* (silenced for ~320 generations in 100% of animals) or wild-type animals. Pink arrowheads indicate the nucleus of the distal tip cell. Numbers within images refer to number of RNAs per 100 µm^2 with standard error of the mean. Animals with median values of fluorescence or RNA signal in the distal region are shown in representative images along with the loop and proximal regions within the same animals. Number of animals imaged per region is indicated within the brightfield image. Positions of smFISH probes within the RNAs is shown in Supplementary Fig. 2d.

c *mCherry*, *gfp* and *tbb-2* pre-mRNA (*left*) or mRNA (*right*) levels were measured by qRT-PCR in animals that express *T* and in animals that showed loss of expression from *T* for >200 generations (*iT*).

d *hrde-1(-)* mutants were mated with *iT* silenced for 171 generations, and scoring was performed in cross progeny, in F2 and F3 descendants.

e Experiment depicting the test for whether *iT* that had been silenced in 100% of animals for 270 generations recovers expression upon removal of *hrde-1* (orange) can show silencing upon re-introduction of *hrde-1(+)* (grey) without re-initiating mating-induced silencing in the descending generations. F3 animals of the genotype *hrde-1(+/-); T/+* from F2 *hrde-1(-); T* hermaphrodites crossed with N2 males were not obtained due to experimental constraints (Supplementary Methods).

f qRT-PCR of mRNA and ChIP-qPCR of H3K9me3 levels of an *hrde-1* target gene[40,41], *R11A8.1*, were measured in wild-type, *hrde-1(-)*, *T* and *iT* animals. H3K9me3 measurements were normalized to wild-type levels. Similar to previous reports, we detected a decrease in H3K9me3 at the *R11A8.1* gene upon loss of HRDE-1, however, no significant change in mRNA was detected. mRNA levels of *R11A8.1* was not significantly altered between *T*, *iT* and wild-type animals and hence was used as a control gene for ChIP experiments. Each dot represents one biological replicate and black line indicates the median value. Each mRNA measurement is the median of five technical replicates.

g H3, H3K9me1, H3K9me2 and H3K9me3 levels were measured at genomic *mCherry* and *gfp* in *T* and *iT* animals. Measurements were normalized to levels at *R11A8.1* measured from each sample's respective input and then to *T*. Each circle represents one biological replicate, which is the median of five technical replicates and black line indicates the median value.

Scoring of silencing (d and e) is as in Fig. 1c. Chromosomes with a *dpy* marker (blue font), number of animals scored (n) and scale bar (10 µm) are indicated. Asterisks indicate $P < 0.05$ and 'ns' indicates $P > 0.05$ using χ^2 test (d) or two-sided Student's t-test (c, f and g). Hash symbol (c) indicates $P = 0.05$ using two-sided Student's t-test. Also see 'Genetic Crosses' in Methods.

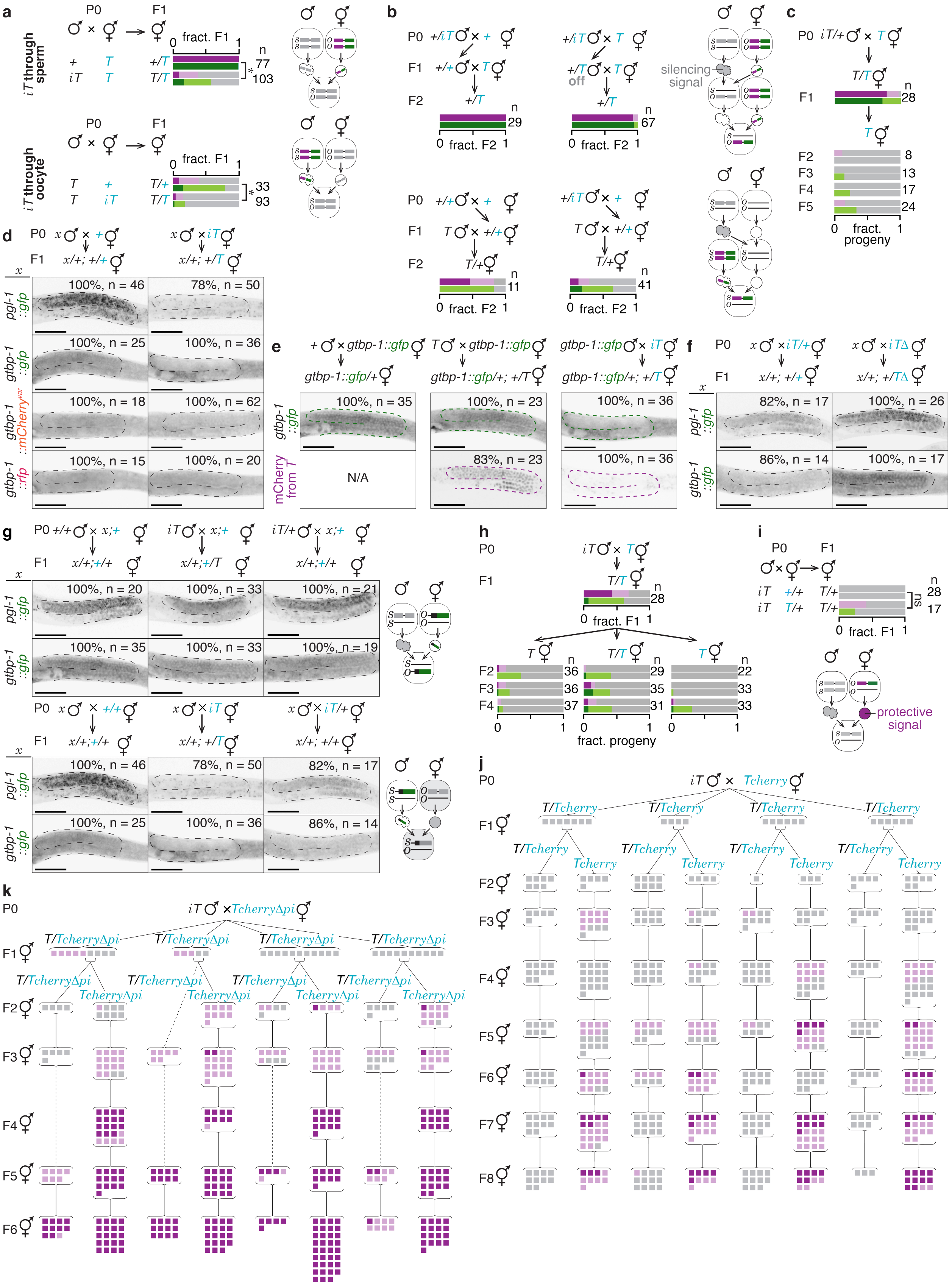
a
iT through sperm
P0
F1
fract. F1
n
+/T
T/T
77
103
*
iT through oocyte
T/+
T/T
33
93
b
P0 +/iT × +
F1 +/+ × T
F2 +/T
29
fract. F2
+/iT × T
+/T off × T
67
silencing signal
P0 +/+ × +
F1 T × +/+
F2 T/+
11
+/iT × +
41
c
P0 iT/+ × T
F1 T/T
28
F2
F3
F4
F5
8
13
17
24
fract. progeny
d
P0 x × +
F1 x/+; +/+
x × iT
x/+; +/T
pgl-1 ::gfp
gtbp-1 ::gfp
gtbp-1 ::mCherry
gtbp-1 ::rfp
100%, n = 46
78%, n = 50
100%, n = 25
100%, n = 36
100%, n = 18
100%, n = 62
100%, n = 15
100%, n = 20
e
+ × gtbp-1::gfp
gtbp-1::gfp/+
T × gtbp-1::gfp
gtbp-1::gfp/+; +/T
gtbp-1::gfp × iT
gtbp-1::gfp/+; +/T
mCherry from T
100%, n = 35
100%, n = 23
100%, n = 36
N/A
83%, n = 23
100%, n = 36
f
P0 x × iT/+
F1 x/+; +/+
x × iTΔ
x/+; +/TΔ
82%, n = 17
100%, n = 26
86%, n = 14
100%, n = 17
g
P0 +/+ × x;+
F1 x/+;+/+
iT × x;+
x/+;+/T
iT/+ × x;+
x/+;+/+
100%, n = 20
100%, n = 33
100%, n = 21
100%, n = 35
100%, n = 33
100%, n = 19
P0 x × +/+
F1 x/+;+/+
x × iT
x/+; +/T
x × iT/+
x/+; +/+
100%, n = 46
78%, n = 50
82%, n = 17
100%, n = 25
100%, n = 36
86%, n = 14
h
P0 iT × T
F1 T/T
28
fract. F1
T
T/T
T
F2
F3
F4
36
36
37
29
35
31
22
33
33
fract. progeny
i
P0
F1
iT +/+ T/+
iT T/+ T/+
28
17
ns
fract. F1
protective signal
j
P0 iT × Tcherry
T/Tcherry
Tcherry
F1
F2
F3
F4
F5
F6
F7
F8
k
P0 iT × TcherryΔpi
T/TcherryΔpi
TcherryΔpi
F1
F2
F3
F4
F5
F6

Supplementary Fig. 7. Variants of *T* can recover from silencing by a heritable silencing signal acting in *trans*.

a Animals expressing *T* were mated with *iT* animals that remained silenced for many generations (*iT* gen. number indicated in Fig. 4b), and cross progeny were scored. The combined data from each cross in Fig. 4b is shown here.

b Crosses to test the transmission of the separable silencing signal across more than one generation.

c Silencing of *T* in *trans* by *iT* was assessed across generations. The remaining results of this cross showing the effect of separable silencing signal is shown in Fig. 4f.

d Males that express homologous (*gfp*) or non-homologous (*rfp* or synonymous *mCherry* variant) sequences fused to endogenous genes (*x* = *pgl-1* or *gtbp-1*) expressed in the germline (*pgl-1*) or expressed ubiquitously (*gtbp-1*) were mated with non-transgenic or *iT* hermaphrodites and fluorescence of PGL-1::GFP, GTBP-1::GFP, GTBP-1::mCherry^{var} or GTBP-1::RFP was imaged in cross progeny. Here, and in the panels below, percentages less than 100 report animals that are silenced as much as or more than the animals shown in the image.

e *gtbp-1::gfp* animals were mated with non-transgenic, *T* or *iT* animals and cross progeny were imaged. Expression level of *mCherry* from *T* is also indicated. N/A, not applicable.

f Males that express *pgl-1::gfp* or *gtbp-1::gfp* were mated with hemizygous *iT* or homozygous *iT*Δ hermaphrodites and GFP fluorescence from the tagged gene was scored in cross progeny that did not inherit *iT* or that inherit *T*Δ. Images from the left panels are also represented for a different comparison in panel **g**.

g Animals that express *pgl-1::gfp* or *gtbp-1::gfp* were mated with homozygous or hemizygous *iT* animals and GFP fluorescence from the tagged gene was scored in cross progeny. Schematics: Maternal (*bottom*) but not paternal (*top*) transmission of the silencing signal (grey filling) affects the expression of homologous genes (green box) in cross progeny, indicating that the hermaphrodite parent carrying *iT* transmits a different type or level of silencing signal (shaded P0 hermaphrodite) compared to the male parent[32,35,38,39].

h *iT* males (silenced upon mating for >250 generations) were mated with *T* hermaphrodites and resulting cross progeny and subsequent generations of descendants of indicated genotypes were scored for expression of mCherry and GFP.

i *Top*, *iT* males were mated with non-transgenic or hemizygous hermaphrodites and cross progeny inheriting only paternal *iT* were scored. *Bottom*, schematic representation of *iT* males mated with hemizygous hermaphrodites indicates the inheritance of both parental protective and silencing signals. The protective signal in this case aids in recovery of weak mCherry and GFP expression from *iT* that remained silenced for ~78 generations.

j and **k** *Tcherry* or *Tcherry*Δ*pi* hermaphrodites were mated with *iT* males and maternally inherited *mCherry* was scored in resulting cross progeny by examining cytoplasmic mCherry fluorescence (i.e. not the nuclear protein-encoding *mCherry::h2b* that is paternally inherited, albeit in a silenced state) in subsequent generations of descendants of indicated genotypes. GFP fluorescence from *iT* was off in all scored animals across generations (data not shown), and therefore independent of the level of mCherry fluorescence from *Tcherry* or *Tcherry*Δ*pi*. Scoring of silencing is as in Fig. 1c. Chromosomes with a *dpy* marker (blue font), number of animals scored (n) and scale bar (50 µm) are indicated. Germlines of representative cross progeny at L4 stage are outlined and percentages of animals with the depicted expression are indicated (d to g). Asterisks indicate $P < 0.05$ and 'ns' indicates $P > 0.05$ using χ^2 test.

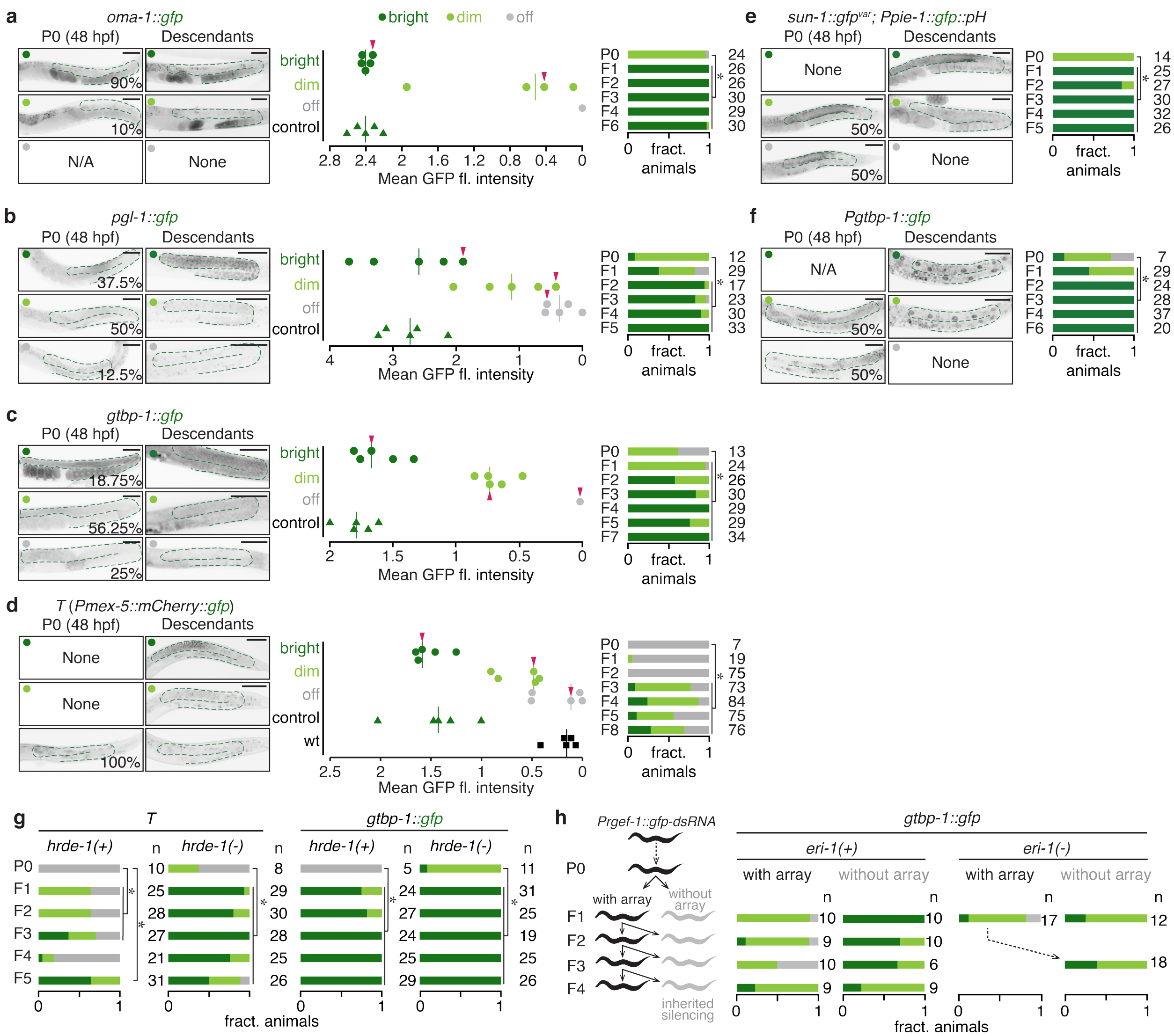

Supplementary Fig. 8. The same target sequence can show variability in transgenerational silencing within the germline upon feeding RNAi.

a to f Six target genes expressing *gfp* (green) were exposed to control RNAi or *gfp* RNAi. The target genes were low copy (*Ppie-1::gfp::pH*, *oma-1::gfp*, *T* (*Pmex-5::mCherry::gfp*)) transgenes or endogenous gene reporters (*gtbp-1::gfp*, *pgl-1::gfp*, *Pgtbp-1::gfp*). Representative images of the germline (*far left*) of P0 animals exposed to RNAi for 24 hours and imaged an additional 24 hours later (48 hours post feeding (hpf)) to eliminate any GFP protein perdurance, are shown. Images of (*middle left*) and the level of GFP expression in (*middle right*) representative descendant animals (F1-F8) categorized as bright, dim or off are shown. Average (green or grey vertical lines) GFP fluorescence intensity within the germline was calculated for descendants of animals exposed to *gfp*-dsRNA (circles, bright: dark green, dim: light green, off: grey) or control dsRNA (green triangles). One to five L4-staged hermaphrodites were quantified after scoring fluorescence from animals by eye within each category. Red arrowheads indicate fluorescence of animals shown in representative images on the left. P0 animals (24 hpf) and F1-F8 descendants were analysed for expression of GFP and categorized based on intensity of fluorescence (*far right*). The P0 to F7 data for *gtbp-1::gfp* (**c**) are the same as in

Fig. 6d. Asterisks indicate $P < 0.05$ using χ^2 test. Scale bar (50 μm) and numbers of animals scored (n) are indicated. N/A indicates not available. Percentages of animals that are silenced as much as or more than the animals shown in the image.

g Hermaphrodites expressing *T* or *gtbp-1::gfp* in a wild-type (*hrde-1(+)*) or *hrde-1(-)* background were exposed to *gfp* RNAi for 24 hours and descendants in subsequent generations (F1-F5) were scored.

h *gtbp-1::gfp* animals expressing neuronal dsRNA against *gfp* (*Prgef-1::gfp-dsRNA*, black) from a mitotically unstable array can have progeny with or without the array. Animals expressing dsRNA in a wild-type (*eri-1(+)*) or *eri-1(-)* background with or without the dsRNA array were scored for expression of GFP. Asterisks indicate $P < 0.05$ using χ^2 test. Numbers of animals scored (n) are indicated.

Supplementary Tables

Supplementary Table 1. Reports on heritability of RNA silencing suggest variable stability of induced RNA silencing.

Target	Genetic background	Generations of inherited silencing	Reference
dpy-11, mex-3, unc-22, lir-1, lin-15, unc-15, dpy-13, sqt-3, dpy-28, pos-1, par-1, dpy-11	*eri-1(-)*	1	42-51
Plet-858::gfp, Psur-5::sur-5::gfp, Pmyo-3::gfp, pes-10::gfp	wild type	1	43, 46, 51, 52
Pdpy-30::mcherry::gpd-2/3::gfp	wild type	1	53
mom-2, pos-1, sgg-1,<br>*unc-22, dpy-11*	wild type	2	54, 55
Ppie-1::gfp::H2B	wild type	3	50
oma-1	*met-2(-); set-25(-); set-32(-)*	2-5	20, 42, 44, 50, 56-58
Ppie-1::gfp::H2B	wild type	1-9	12, 20, 42, 51, 58-60
Pcdk-1::gfp	wild type	> 10	16
Ppie-1::gfp::H2B	*eri-1(-)*	>20	61
Ppie-1::gfp::H2B	*heri-1(-)*	>23	56
gfp::his-58	wild type	>30	14
oma-1<br>*Pmex-5::gfp*	*met-2(-)*	>10<br>>30	57, 62
Pmex-5::mCherry::gfp	wild type	>25	63

Supplementary Table 2. Comparison of mating-induced silencing with related epigenetic phenomena.

Phenomenon	Reference(s) for the phenomenon	Similarity with mating-induced silencing	Difference from mating-induced silencing
Paramutation in plants, flies, or mice	1, 2, 4-9	Silencing is transgenerational. Silenced allele inherited through either gamete can silence homologous sequences.	Whether silencing will occur is not predictable at the population level. When a silenced allele induces meiotically heritable silencing of another allele, this allele also becomes a silencing allele.
RNA induced epigenetic silencing (RNAe)	12-14, 16, 19	Initiation requires PRG-1; maintenance requires HRDE-1. Silencing is transgenerational.	Silencing cannot be predictably initiated. The same DNA inserted into the same locus can show expression or silencing.

			Changes upon mating, if any, are unknown.
Multi-generational RNAe caused by meiotic silencing by unpaired DNA	27	Initiation requires PRG-1. oxSi487 (T in our study) introduced through the male parent showed silencing in cross progeny.	Effect of introducing oxSi487 through the hermaphrodite parent on silencing in cross progeny or its hemizygous descendants was not tested.
RNA-induced epigenetic gene activation (RNAa)	17, 18, 64	Extragenic signal can be inherited from male to control gene expression in progeny. Inheritance of an active transgene from hermaphrodite affects expression of paternally inherited transgene.	Extragenic signals inherited from sperm promote expression.
Meiotic silencing by unpaired DNA	28	Silencing occurs on hemizygous DNA.	DNA must be unpaired during meiosis for silencing.
Epigenetic licensing of *fem-1*	26	Maternal transcript of a gene is sufficient to enable expression of the paternal copy in the zygote.	Repeated crossing was required for increased severity of silencing.
Genomic imprinting and parent of origin effects	38, 39, 65	Silencing occurs when a gene is inherited through a specific gamete.	Expression is reset upon passage through the other gamete.
Transposon silencing in flies	6, 66	Inherited piRNAs silence a paternally inherited gene.	Maternal transcript does not prevent gene silencing.
Transvection in flies	67	Interaction between alleles on homologous chromosomes can result in changed expression.	Changes in gene expression are not heritable.
Licensing by DNA sequences	25	Not all transgenes are susceptible to germline silencing.	Initiation of silencing is independent of mating.

Supplementary Table 3. Strains used*.

Strain Name	Genotype
N2	wild type
AMJ471	*jamEx140* [*Prgef-1::gfp-dsRNA:: unc-54 3' utr & Pmyo-2::DsRed::unc-54 3' utr*]
AMJ501	*oxSi487* (*Pmex-5::mCherry::h2b::tbb-2 3'utr::gpd-2 operon::gfp::h2b::cye-1 3' utr* + *unc-119(+)*) II; *unc-119(ed3)* III?; *sid-1(qt9)* V
AMJ506	*prg-1(tm872)* I; *oxSi487* II; *unc-119(ed3)*? III
AMJ544	*oxSi487* II; *unc-119(ed3)?* III; *nrde-3(tm1116)* X
AMJ545	*oxSi487* II; *unc-119(ed3)* III?; *rde-1(ne219)* V
AMJ552	*oxSi487 dpy-2(jam33)* II; *unc-119(ed3)?* III [*iT*]
AMJ577	*hrde-1(tm1200)* III [4x]
AMJ581	*oxSi487 dpy-2(e8)* II
AMJ586	*oxSi487 dpy-2(e8)* II; *unc-119(ed3)?* III; *rde-1(ne219)* V
AMJ587	*rde-3/mut-2(jam9)* I
AMJ591	*jamSi25* [*Punc-119deletion *jamSi19*] II [*T*ΔΔ]
AMJ593	*oxSi487 dpy-2(e8)* II; *unc-119(ed3)?* III; *sid-1(qt9)* V
AMJ602	*oxSi487 dpy-2(e8)* II; *unc-119(ed3)? hrde-1(tm1200)* III
AMJ626	*rrf-1(ok589)* I; *oxSi487 dpy-2(e8)* II; *unc-119(ed3)?* III
AMJ646	*dpy-17(e164) unc-32(e189)* III; *rde-1(ne219)* V
AMJ647	*dpy-17(e164) unc-32(e189)* III; *sid-1(qt9)* V
AMJ667	*dpy-20(e1282) ax2053* [*gtbp-1::gfp*] IV
AMJ673	*rrf-1(ok589)* I; *dpy-2(e8) unc-4(e120)* II
AMJ675	*oxSi487* II; *unc-119(ed3)? hrde-1(tm1200)* III
AMJ683	*oxSi487 dpy-2(e8)* II; *unc-119(ed3)?* III; *nrde-3(tm1116)* X
AMJ685	*K08F4.2::gfp* [*Pgtbp-1::gtbp-1::gfp*] IV; *jamEx140*
AMJ689	*rrf-1(ok589)* I; *oxSi487* II; *unc-119(ed3)?* III
AMJ690	*dpy-2(e8) unc-4(e120)* II; *nrde-3(tm1116)* X
AMJ691	*dpy-2(e8) unc-4(e120)* II; *hrde-1(tm1200)* III
AMJ692	*oxSi487 dpy-2(e8)* II [*iT*]
AMJ693	*dpy-2(e8) unc-4(e120)* II; *Pmex-5::mCherry^{var2}::mex-5::mex-5 3' utr* IV
AMJ709	*dpy-10(jam21) jamSi25* [*Punc-119deletion *jamSi19*] II [*T*ΔΔ]
AMJ711	*prg-1(tm872)* I [1x]
AMJ712	*dpy-2(e8) unc-4(e120)* II; *Pgtbp-1::gtbp-1::RFP::linker::3xflag::gtbp-1 3'utr* IV
AMJ713	*dpy-2(e8) unc-4(e120)* II; *Ppgl-1::pgl-1::gfp::pgl-1 gfp 3' utr* IV
AMJ714	*oxSi487* II; *unc-119(ed3)? hrde-1(tm1200)* III
AMJ724	*oxSi487* II; *unc-119(ed3)?* III [*iT*]
AMJ725	*oxSi487* II; *unc-119(ed3)?* III
AMJ727	*dpy-2(e8) unc-4(e120)* II; *gtbp-1::mCherry^{var}* IV
AMJ753	*dpy-10(jam38) oxSi487* II; *unc-119(ed3)* III
AMJ763	*dpy-10(jam40) jamSi16* [*Pmex-5::mCherry::h2b::cye-1 3' utr *oxSi487*] II [*T*Δ]

AMJ765	*dpy-10(jam41) jamSi18* [*Pmex-5::mCherry::h2b::cye-1 3' utr *oxSi487*] II [*T*Δ]
AMJ766	*jamSi19* [*Pmex-5::mCherry::h2b::cye-1 3' utr *oxSi487*] II [*T*Δ]
AMJ767	*dpy-10(jam42) jamSi20* [*Pmex-5::mCherry::h2b::cye-1 3' utr *oxSi487*] II [*T*Δ]
AMJ768	*dpy-10(jam43) jamSi21* [*Pmex-5::mCherry::h2b::cye-1 3' utr *oxSi487*] II [*T*Δ]
AMJ769	*dpy-10(jam44) oxSi487* II; *unc-119(ed3)* III
AMJ774	*dpy-10(jam139) jamSi23* [*Pmex-5::mCherry (6 bp indel)::h2b::tbb-2 3' utr::gpd-2 operon::gfp::h2b::cye-1 3' utr *oxSi487*] II; *unc-119(ed3)* III [*T**]
AMJ777	*dpy-10(jam45)* II
AMJ792	*dpy-10(jam46)* II
AMJ819	*Pgtbp-1::gtbp-1::gfp eri-1(mg366)* IV
AMJ842	*Pgtbp-1::gtbp-1::gfp eri-1(mg366)* IV; *jamEx140*
AMJ844	*oxSi487 dpy-2(e8)* II [*iT*]
AMJ917	*dpy-10(jam47) jamSi20* [*Pmex-5::mCherry::h2b::cye-1 3' utr *oxSi487*] II; *unc-119(ed3)* III [*iT*Δ]
AMJ918	*dpy-10(jam140) jamSi32* [*Pmex-5::mCherry (3 bp indel)::h2b::cye-1 3' utr *jamSi19*] II; *unc-119(ed3)* III [*T*Δ*]
AMJ919	*dpy-10(jam141) jamSi33* [*Pmex-5::mCherry (2 bp indel)::h2b::cye-1 3' utr *jamSi25*] II; *unc-119(ed3)* III [*T*ΔΔ*]
AMJ922	*prg-1(tm872)* I [1x]; *dpy-2(e8) oxSi487* II; *unc-119(ed3)?* III
AMJ923	*prg-1(tm872)* I [1x]; *dpy-2(e8) unc-4(e120)* II
AMJ926	*dpy-10(jam39) jamSi27* [*Pmex-5::mCherry::cye-1 3' utr *jamSi25*] II [*T*ΔΔΔ]
AMJ928	*jamSi27* [*Pmex-5::mCherry::cye-1 3' utr *jamSi25*] II [*T*ΔΔΔ]
AMJ930	*dpy-10(jam68)* II
AMJ929	*oxSi487* II
AMJ1045	*oxSi487* II; *unc-119(ed3)? hrde-1(tm1200)* III
AMJ1100	*oxSi487 unc-4(e120)* II; *unc-119(ed3)?* III
AMJ1101	*oxSi487 unc-4(e120)* II; *unc-119(ed3)?* III
AMJ1102	*oxSi487 unc-4(e120)* II; *unc-119(ed3)?* III
AMJ1103	*oxSi487 unc-4(e120)* II; *unc-119(ed3)?* III
AMJ1116	*oxSi487 dpy-2(e8)* II; *unc-119(ed3)?* III; *met-2(n4256)* III
AMJ1117	*oxSi487 dpy-2(e8)* II; *unc-119(ed3)?* III; *met-2(n4256)* III
AMJ1118	*oxSi487 dpy-2(e8)* II; *unc-119(ed3)?* III; *met-2(n4256)* III
AMJ1126	*mut-16(pk710)* I; *oxSi487 dpy-2(e8)* II; *unc-119(ed3)?* III
AMJ1127	*mut-16(pk710)* I; *oxSi487 dpy-2(e8)* II; *unc-119(ed3)?* III
AMJ1128	*mut-16(pk710)* I; *oxSi487 dpy-2(e8)* II; *unc-119(ed3)?* III
AMJ1135	*rde-3/mut-2(jam9)* I; *oxSi487 dpy-2(e8)* II; *unc-119(ed3)?* III
AMJ1136	*rde-3/mut-2(jam9)* I; *oxSi487 dpy-2(e8)* II; *unc-119(ed3)?* III
AMJ1137	*met-2(n4256)* III; *Pgtbp-1::gtbp-1::gfp* IV
AMJ1138	*met-2(n4256)* III; *Pgtbp-1::gtbp-1::gfp* IV
AMJ1139	*met-2(n4256)* III; *Pgtbp-1::gtbp-1::gfp* IV
AMJ1142	*oxSi487 dpy-2(e8)* II; *unc-119(ed3)?* III; *pgl-1(ct131) him-3(e1147)* IV
AMJ1143	*oxSi487 dpy-2(e8)* II; *unc-119(ed3)?* III; *pgl-1(ct131) him-3(e1147)* IV

AMJ1157	*oxSi487 dpy-2(jam33)* II; *unc-119(ed3)?* III; *rde-8(jam75)* IV
AMJ1158	*oxSi487 dpy-10(jam82) dpy-2(jam33)* II; *unc-119(ed3)?* III; *rde-8(jam76)* IV
AMJ1162	*dpy-10(jam43) oxSi487* II; *unc-119(ed3)* III
AMJ1170	*jamSi37* [*Pmex-5::mCherry::cye-1 3'UTR + unc-119(+)*] II; *unc-119(ed3)* III
AMJ1174	*dpy-10(jam106) jamSi37 [Pmex-5::mCherry::cye-1 3'UTR]* II*; unc-119(ed3)* III
AMJ1176	*jamSi27* II*; Pgtbp-1::gtbp-1::gfp* IV
AMJ1186	*jamSi37* II*; unc-119(ed3)?* III
AMJ1190	*jamSi38* [*Pmex-5::mCherry::cye-1 3'utr*] II; *unc-119(ed3)* III [*Tcherry^{Crispr}*]
AMJ1191	*jamSi40* [*Pmex-5::mCherry::cye-1 3'utr*] II; *unc-119(ed3)* III [*Tcherry^{Crispr}*]
AMJ1192	*jamSi41* [*Pmex-5::mCherry::cye-1 3'utr*] II; *unc-119(ed3)* III [*Tcherry^{Crispr}*]
AMJ1195	*jamSi59* [*Pmex-5::gfp::cye-1 3'UTR + unc-119(+)*] II*; unc-119(ed3)* III [*Tgfp*]
AMJ1200	*jamSi60* [*Pmex-5::gfp::cye-1 3'UTR + unc-119(+)*] II*; unc-119(ed3)* III [*Tgfp*]
AMJ1206	*set-32(jam46)* I*; oxSi487 dpy-2(e8)* II*; unc-119(ed3)?* III
AMJ1207	*oxSi487 dpy-2(e8) heri-1(jam47)* II*; unc-119(ed3)?* III
AMJ1208	*jam197* [*Pmex-5::mCherry::mex-5::mex-5 3'UTR*] IV
AMJ1209	*jamSi39* [*Pmex-5::mCherry (without piRNA sites)::cye-1 3' utr*] II; *unc-119(ed3)* III [*TcherryΔpi*]
AMJ1210	*jamSi42* [*Pmex-5::mCherry (without piRNA sites)::cye-1 3' utr*] II; *unc-119(ed3)* III [*TcherryΔpi*]
AMJ1211	*jamSi43* [*Pmex-5::mCherry (without piRNA sites)::cye-1 3' utr*] II; *unc-119(ed3)* III [*TcherryΔpi*]
AMJ1212	*jamSi44* [*Pmex-5::mCherry (without piRNA sites)::cye-1 3' utr*] II; *unc-119(ed3)* III [*TcherryΔpi*]
AMJ1213	*dpy-10(jam73) jamSi39* II; *unc-119(ed3)* III [*TcherryΔpi*]
AMJ1214	*dpy-10(jam74) jamSi42* II; *unc-119(ed3)* III [*TcherryΔpi*]
AMJ1215	*dpy-10(jam84) jamSi43* II; *unc-119(ed3)* III [*TcherryΔpi*]
AMJ1216	*dpy-10(jam85) jamSi44* II; *unc-119(ed3)* III [*TcherryΔpi*]
AMJ1228	*mut-16(pk710)* I; *oxSi487* II; *unc-119(ed3)* III
AMJ1236	*jamSi37* II*; unc-119(ed3?)* III*; Pgtbp-1::gtbp-1::gfp* IV
AMJ1238	*dpy-10(jam106) jamSi37* II
AMJ1240	*dpy-10(jam106) jamSi37* II*; ccTi1594 [Pmex-5::gfp::gpr-1::smu-1 3'UTR + Cbr-unc-119(+)] unc-119(ed3?)* III
AMJ1245	*jamSi61 [Pmex-5::gfp::cye-1 3' utr + unc-119(+)]* II; *unc-119(ed3)* III [*Tgfp*]
AMJ1248	*dpy-10(jam142) jamSi51* [*Pmex-5::cye-1 3' utr *jamSi37*] II; *unc-119(ed3)* III [*TΔorf*]
AMJ1249	*dpy-10(jam143) jamSi49* [*Pmex-5::cye-1 3' utr *jamSi37*] II; *unc-119(ed3)* III [*TΔorf*]
AMJ1259	*hrde-1(tm1200)* III; *fog-2(q71)* V
AMJ1260	*hrde-1(tm1200)* III; *fog-2(q71)* V
AMJ1261	*hrde-1(tm1200)* III; *fog-2(q71)* V
AMJ1267	*dpy-10(jam106) jamSi37* II*; ccTi1594 unc-119(ed3?)* III
AMJ1268	*dpy-10(jam106) jamSi37* II*; ccTi1594 unc-119(ed3?)* III
AMJ1272	*jamSi45* [*unc-119(+) Pmex-5::mCherry::mex-5 3' utr*] II; *hrde-1(tm1200)* III [*Tcherry mex-5 3' utr*]

AMJ1273	*jamSi47* [*unc-119(+) Pmex-5::mCherry::mex-5 3' utr*] II; *hrde-1(tm1200)* III [*Tcherry mex-5 3' utr*]
AMJ1274	*jamSi46* [*unc-119(+) Pmex-5::mCherry::mex-5 3' utr*] II; *hrde-1(tm1200)* III [*Tcherry mex-5 3' utr*]
AMJ1275	*jamSi48* [*unc-119(+) Pmex-5::mCherry::mex-5 3' utr*] II; *hrde-1(tm1200)* III [*Tcherry mex-5 3' utr*]
AMJ1288	*dpy-10(jam144) jamsSi52* II; *unc-119(ed3)* III [*TcherryΔpi N*]
AMJ1290	*dpy-10(jam146) jamsSi54* II; *unc-119(ed3)* III [*TcherryΔpi C*]
AMJ1296	*unc-119(ed3) cde-1(jam111)* III
AMJ1307	*oxSi487* II*; unc-119(ed3) cde-1(jam110)* III
AMJ1308	*oxSi487 dpy-10(jam138)* II*; unc-119(ed3)? cde-1(jam111)* III
AMJ1320	*rrf-1(ok589) ego-1(jam93)* I
AMJ1321	*rrf-1(ok589) ego-1(jam93)* I
AMJ1336	*dpy-10(jam147) jamSi57* [*Pmex-5::mCherry(exon 4)::cye-1 3' utr *jamSi39*] II; *unc-119(ed3)* III [*TcherryΔpi exon 4*]
AMJ1337	*dpy-10(jam149) jamSi58* [*Pmex-5::mCherry(exon 4)::cye-1 3' utr *jamSi39*] II; *unc-119(ed3)* III [*TcherryΔpi exon 4*]
AMJ1338	*jamSi56* I; *unc-119(ed3)* III [*Tcherry* I]
AMJ1339	*jamSi63* [*unc-119(+) Pmex-5::mCherry::tbb-2 3' utr*] II; *hrde-1(tm1200)* III [*Tcherry tbb-2 3' utr*]
AMJ1340	*jamSi64* [*unc-119(+) Pmex-5::mCherry::tbb-2 3' utr*] II; *hrde-1(tm1200)* III [*Tcherry tbb-2 3' utr*]
AMJ1341	*jamSi65* [*unc-119(+) Pmex-5::mCherry::tbb-2 3' utr*] II; *hrde-1(tm1200)* III [*Tcherry tbb-2 3' utr*]
DR439	*unc-8(e49) dpy-20(e1282)* IV
EG4322	*ttTi5605* II; *unc-119(ed9)* III
EG6787	*oxSi487* II*; unc-119(ed3)* III
EG6771	*oxSi466* [*Pdpy-30::gfp::h2b::tbb-2 cb-unc-119(+)*] II; *unc-119(ed3)* III [gift from Christian Frøkjær-Jensen]
EG6779	*oxSi474* [*Pdpy-30::gfp::h2b::tbb-2 cb-unc-119(+)*] I; *unc-119(ed3)* III [gift from Christian Frøkjær-Jensen]
EG6808	*unc-119(ed3)* III; *oxTi132* [*Pdpy-30::gfp::h2b::tbb-2 cb-unc-119(+)*] V (*him-5* in background?) [gift from Christian Frøkjær-Jensen]
EG6810	*unc-119(ed3)* III; *oxTi134* [*Pdpy-30::gfp::h2b::tbb-2 cb-unc-119(+)*] I (*him-5* in background?) [gift from Christian Frøkjær-Jensen]
EG6814	*unc-119(ed3)* III; *oxTi138* [*Pdpy-30::gfp::h2b::tbb-2 cb-unc-119(+)*] I (*him-5* in background?) [gift from Christian Frøkjær-Jensen]
EG6838	*unc-119(ed3) oxTi162* [*Pdpy-30::gfp::h2b::tbb-2 cb-unc-119(+)*] III (*him-5* in background?) [gift from Christian Frøkjær-Jensen]
GE1708	*dpy-2(e8) unc-4(e120)* II
GR1373	*eri-1(mg366)* IV
HC196	*sid-1(qt9)* V
HC780	*rrf-1(ok589)* I
HT1593	*unc-119(ed3)* III
JH3197	*ax2053* (*Pgtbp-1::gtbp-1::gfp*) IV [gift from Geraldine Seydoux]

JH3270	*Ppgl-1::pgl-1::gfp::pgl-1 gfp 3' utr* IV [gift from Geraldine Seydoux]
JH3296	*Pmex-5::mCherry*^{var2}*::mex-5::mex-5 3' utr* IV [gift from Geraldine Seydoux]
JH3323	*Pgtbp-1::gtbp-1::mCherry::gtbp-1 3' utr* IV [*gtbp-1::mCherry*^{var}; gift from Geraldine Seydoux]
JH3337	*Pgtbp-1::gtbp-1::RFP::linker::3xflag::gtbp-1 3'utr* II [gift from Geraldine Seydoux]
JH4009	*Pgtbp-1::gfp::h2b::gtbp-1 3'utr* II [gift from Geraldine Seydoux]
MT13293	*met-2(n4256)* III
NL1810	*mut-16(pk710)* I
OCF62	*jfSi1* [*Psun-1::gfp cb-unc-119(+)*] II*; ltIs38* [*(pAA1) pie-1::GFP::PH(PLC1delta1) + unc-119(+)*] [*sun-1::gfp*^{var}; gift from Orna Cohen-Fix]
OCF69	*ocfSi1* [*Pmex-5::Dendra2::his-58::tbb-2 3' utr + unc-119(+)*] I*; unc-119(ed3)* III [gift from Orna Cohen-Fix]
PD1594	*ccTi1594 unc-119(ed3)* III (*gpr-1 oe*)
SP471	*dpy-17(e164) unc-32(e189)* III
SS2	*pgl-1(ct131) him-3(e1147)* IV
TX189	*unc-199(ed3)* III*; teIs1* [*(pRL475) oma-1p::oma-1::GFP + (pDPMM016) unc-119(+)*] IV
WM27	*rde-1(ne219)* V
WM156	*nrde-3(tm1116)* X
WM161	*prg-1(tm872)* I

*All strains with fluorescent reporters showed invariable expression of fluorescence, except as indicated throughout the manuscript and in OCF69, which showed suppression of expression in one of the 34 animals examined by imaging.

Supplementary Table 4. Oligonucleotides used*.

P1	ATAAGGAGTTCCACGCCCAG
P2	CTAGTGAGTCGTATTATAAGTG
P3	TGAAGACGACGAGCCACTTG
P4	ATCGTGGACGTGGTGGTTAC
P5	CTCATCAAGCCGCAGAAAGAG
P6	GGTTCTTGACAGTCCGAACG
P7	ACGGTGAGGAAGGAAAGGAG
P8	ACAAGAATTGGGACAACTCCAG
P9	AGTAACAGTTTCAAATGGCCG
P10	TCTTCACTGTACAATGTGACG
P11	CACTATTCACAAGCATTGGC
P12	CGGACAGAGGAAGAAATGC
P13	TGCCATCGCAGATAGTCC
P14	TGGAAGCAGCTAGGAACAG
P15	CCGTGACAACAGACATTCAATC
P16	ACGATCAGCGATGAAGGAG
P17	GGAGATCCATGATTAGTTGTGC
P18	GCAGGCATTGAGCTTGAC

P19	TCATCTCGGTACCTGTCGTTG
P20	AGAGGCGGATACGGAAGAAG
P21	CATAACCGTCGCTTGGCAC
P22	TCGAGTCGTGGTACAGATCG
P23	CATGCTCGTCGTAATGCTCG
P24	CGATCGTGCCAGAACAATCC
P25	ATGAAAGCCGAGCAACAACG
P26	AGAATGATGAGTCGCCACAGG
P27	CATGCACAACAAAGCCGACTAC
P28	TGAGAATACGGTCGCAGTTAGG
P29	ACGGATGCCTAGTTGCATTG
P30	CCTTCCCAGAGGGATTCAAGTG
P31	TCTGTTCCTATTCTGTCTGCAC
P32	CGCGGTTCGCAATAGGTTTC
P33	TCACCTAGTCTGTGCCATTTC
P34	TGCGGGTTTCTGTTAGCTTC
P35	GCACAGACTAGGTGAAAGAGAG
P36	ACCTCCCACAACGAGGATTAC
P37	TGGGCGTGGAACTCCTTATC
P38	GGCGAAGAGCAAAGCAGAG
P39	GGGCCGTTATCCTTTCAAATGC
P40	CATGGGCCACGGATTGTAAC
P41	ACGCATCTGTGCGGTATTTC
P42	ATTTAGGTGACACTATAGGATCAGGTAGTGGCCCACCAGTTTTAGAGCTA GAAATAGCAAG
P43	AAAAGCACCGACTCGGT
P44	ATGGTCTCCAAGGGAGAGGAG
P45	GAATCCTATTGCGGGTTATTTTAGCCACTACCTGATCCCTTG
P46	ATTTAGGTGACACTATAGGTGTAATCCTCGTTGTGGGGTTTTAGAGCTAGA AATAGCAAG
P47	CAAGGGATCAGGTAGTGGCTAAAATAACCCGCAATAGGATTC
P48	TAAGGAGTTCCACGCCCAG
P49	TTTCGCTGTCCTGTCACACTC
P50	CGATGATAAAAGAATCCTATTGCGGGTTATTTTTTGAGCCTGCTTTTTTGTA CAAACTTG
P51	CAAGTTTGTACAAAAAAGCAGGCTCAAAAAATAACCCGCAATAGGATTCT TT TATCATCG
P52	AGCTAACAGAAACCCGCATAC
P53	CCTGTCACACTCGCTAAAAACAC
P54	ACAGAAACCCGCATACTCG
P55	ATTTAGGTGACACTATAGATTCCTTGTTCGGTGCTTGGGTTTTAGAGCTAG AAATAGCAAG
P56	ATTCCATGATGGTAGCAAACTCACTTCGTGGGTTTTCACAACGGCAAAATA TCAGTTTTT

P57	ATTTAGGTGACACTATAGCTACCATAGGCACCACGAGGTTTTAGAGCTAG AA ATAGCAAG
P58	CACTTGAACTTCAATACGGCAAGATGAGAATGACTGGAAACCGTACCGCA TG CGGTGCCTATGGTAGCGGAGCTTCACATGGCTTCAGACCAACAGCCTA
P59	ATTTAGGTGACACTATAGACAAATGCCCGGGGGATCGGGTTTTAGAGCTA GAAATAGCAAG
P60	TGAGGTCAAGACCACCTACAAG
P61	GAATCCTATTGCGGGTTATTTTACTTGCTGGAAGTGTACTTGG
P62	CCAAGTACACTTCCAGCAAGTAAAATAACCCGCAATAGGATTC
P63	GACCACCTACAAGGCTAAGAAG
P64	ATTTAGGTGACACTATAGGGGAGAGGGAAGACCATACGGTTTTAGAGCTA GAAATAGCAAG
P65	GCAAAAATTCCCCGACTTTCCC
P66	GAAAAGTTCTTCTCCTTTACTCATTTTTGAGCCTGCTTTTTTGTAC
P67	GTACAAAAAAGCAGGCTCAAAAATGAGTAAAGGAGAAGAACTTTTC
P68	CCCATGGAACAGGTAGTTTTCC
P69	CGACTTTCCCCAAAATCCTGC
P70	ACAGGTAGTTTTCCAGTAGTGC
P71	AGAGGGATTCAAGTGGGAGAG
P72	TGGGTCTTACCGCGTATACC
P73	TGATCCCTTGTAAAGCTCATCC
P74	GTGTGTGCTGCTCGGTTAAG
P75	AATTCCACAGTTGCTCCGAC
P76	TCATCTCGCCCGATTCATTG
P77	CCGTTTCTTCCTGGTAATCC
P78	GGGTGAAGGTGATGCAACATAC
P79	GGGACAACCTGTGTGCATG
P80	AAGGTCCACATGGAGGGATC
P81	AAAGTAATTCTACAGTATTCCTGAGATG
P82	CGTCTCTTGATATTCCTTGC
P83	CCAAGCGAATGGAAGCTGAAAATT
P84	CAAGCGAATGGAAGTGGTCCT
P85	GTAGTGACAAGTGTTGGCCATGG
P86	TCACATACACATCTTCTGCACC
P87	TTGGTAGAAGCTGCATCACTTT
P88	CCAGACGGAACCTTCAAG
P89	TCCGTCTGAAAAAAATTTAATTAATT
P90	GAGATTCAAGGTCCACATGGAGG
P91	ATGGAAGTGGTCCTCCCTTGG
P92	TCTTCGGCGCTAATCTTTTC
P93	CACGAGTTCGAGATCGAG
P94	GTCATCTCCGACGAGCAC
P95	TTCCGTTGTTGGCTTCGTTG
P96	TTCTGTCAGTGGAGAGGG
P97	GTGTTGGCTGAAAATTTAAATAAT

P98	GGTGATGTTAATGGGCAC
P99	TGTTGGCCATGGAACAGG
P100	ATTTAGGTGACACTATAGGATTACTCATAATGACATGGTTTTAGAGCTAGA AATAGCAAG
P101	GGACCACGTGGAGTTCCAGGACATCCAGGTTTTCCAGGTGACCCAGGAGA GTATGGAATT
P102	ATTTAGGTGACACTATAGCGTTGGTGATGGTGATGAGGTTTTAGAGCTAGA AATAGCAAG
P103	ATCTGATTATTATATTTCAGATTACTCATAATTAATGTATTCAATTTGTTAA TATATTTC
P104	ATTTAGGTGACACTATAGTGCTTCGATAGATCTCGAGGTTTTAGAGCTAGA AATAGCAAG
P105	ATTTAGGTGACACTATAGTTCAGCTTACAATGGACTAGTTTTAGAGCTAGA AATAGCAAG
P106	TTAATTCTTAACAAAAAACTGTTTCCGCTCCTACGGATACAACTACATGAA AAATCATCT
P107	ATTTAGGTGACACTATAGAGTAGTTACTGATGAGCTGGTTTTAGAGCTAGA AATAGCAAG
P108	ATTTAGGTGACACTATAGTCGAGCTGTAGGCTCTTGGGTTTTAGAGCTAGA AATAGCAAG
P109	GAGAGATTCAAAAGAACAAAAAAGCCGCAGAGAGCCTACAGCTCGATCT GTAGAGTGTTT
P110	GCUACCAUAGGCACCACGAGGUUUUAGAGCUAUGCU
P111	AGCAUAGCAAGUUAAAAUAAGGCUAGUCCGUUAUCAACUUGAAAAAGU GGCACCGAGUCGGUGCUUU
P112	TGATGATAGCCATGTTATCC
P113	GTGGACCTTGAATCTCATGA
P114	CTCTCCCTCGATCTCGAACTCGTGTC
P115	CTTGGTGACCTTAAGCTTAG
P116	GATATCCCAAGCGAATGGAA
P117	CGTACATGAACTGTGGGGAA
P118	TGCTTGACGTAAGCCTTGGA
P119	GGTAATCTGGGATATCAGCT
P120	GAATCCCTCTGGGAAGGAAA
P121	ATCCTCGAAGTTCATGACTC
P122	GAATCCTGGGTGACGGTGAC
P123	ATGAACTCTCCATCCTGAAG
P124	TCCTCTAAGCTTGACCTTGT
P125	GTCCATCGGATGGGAAGTTG
P126	ATGGTCTTCTTCTGCATGAC
P127	TACATTCTCTCGGAGGAAGC
P128	CTTGATCTCTCCCTTAAGAG
P129	TCCATCCTTAAGCTTAAGTC
P130	TTGACCTCAGCATCGTAGTG
P131	CTTCTTAGCCTTGTAGGTGG

P132	TAAGCTCCTGGAAGCTGGAC
P133	ATCAAGCTTGATGTTGACGT
P134	TGTAATCCTCGTTGTGGGAG
P135	CTCTCGTACTGCTCGACGAT
P136	TTGTAAAGCTCATCCATTCC
P137	AAGTTCTTCTCCTTTACTCA
P138	GAATTGGGACAACTCCAGTG
P139	CCCATTAACATCACCATCTA
P140	CCTCTCCACTGACAGAAAAT
P141	GTAAGTTTTCCGTATGTTGC
P142	TGGAACAGGTAGTTTTCCAG
P143	GGTATCTCGAGAAGCATTGA
P144	TCATGCCGTTTCATATGATC
P145	GGGCATGGCACTCTTGAAAA
P146	TTCTTTCCTGTACATAACCT
P147	GTTCCCGTCATCTTTGAAAA
P148	CCTTCAAACTTGACTTCAGC
P149	ACCTTTTAACTCGATTCTAT
P150	GTGTCCAAGAATGTTTCCAT
P151	GTGAGTTATAGTTGTATTCC
P152	GTCTGCCATGATGTATACAT
P153	CTTTGATTCCATTCTTTTG
P154	CCATCTTCAATGTTGTGTCT
P155	ATGGTCTGCTAGTTGAACGC
P156	CGCCAATTGGAGTA TTTTGT
P157	GTCTGGTAAAAGGACAGGGC
P158	AAGGGCAGATTGTGTGGACA
P159	TCTTTTCGTTGGGATCTTTC
P160	TCAAGAAGGACCATGTGGTC
P161	AATCCCAGCAGCTG TTACAA
P162	TATAGTTCATCCATGCCATG
P163	ATTTAGGTGACACTATAGTCAACTTCTAATTTTAATTCGTTTTAGAGCTAG AAATAGCAAG
P164	AUUUAGGUGACACUAUAGGUGAUGAACUUCGAGGAUGGGUUUUAGAGC UAGAAAUAGCAAG
P165	AUUUAGGUGACACUAUAGCUUUACAAGGGAUCAGGUAGGUUUUAGAGC UAGAAAUAGCAAG
P166	AUUUAGGUGACACUAUAGAAAAAUGGUCUCCAAGGGAGGUUUUAGAGC UAGAAAUAGCAAG
P167	AUUUAGGUGACACUAUAGCCUUCCCAGAGGGAUUCAAGGUUUUAGAGC UAGAAAUAGCAAG
P168	TCTCCTTCCCAGAGGGATTCAAGTGGGAGAGAGTGTAAAATAACCCGCAA TAGGATTCTTT TATCATCGA
P169	CAGAGACAAGTTTGTACAAAAAAGCAGGCTCAAAAATGAACTTCGAGGAT GGAGGAGTCGTCACCGTCAC

P170	AUUUAGGUGACACUAUAGAAUGGUCUCCAAGGGAGAGGGUUUUAGAGC UAGAAAUAGCAAG
P171	AUUUAGGUGACACUAUAGCUUUACAAGGGAUCAGGUAGGUUUUAGAGC UAGAAAUAGCAAG
P172	CAGAGACAAGTTTGTACAAAAAAGCAGGCTCAAAAAATAACCCGCAATA GGATTCTTTTATCATCGAAAT
P173	AUUUAGGUGACACUAUAGAAAAAUGGUCUCCAAGGGAGGUUUUAGAGC UAGAAAUAGCAAG
P174	AUUUAGGUGACACUAUAGUAAUCUGAUUUAAAUUUUCAGUUUUAGAGC UAGAAAUAGCAAG
P175	AGACAAGTTTGTACAAAAAAGCAGGCTCAAAAATGGGACACTACGATGCT GAGGTCAAGACCACCTACAA
P176	GCACATACTTTCCGTCTGAAAAAAT
P177	CGTGGCACATACTTTCCGTTGTTG
P178	GAAAGTAGTGACAAGTGTTGGCTG
P179	GGAAGCTGAAAATTTAAATAATCAG
P180	UUUCAGACAGAGAAUGAAAG
P181	ATTAATTTTATCGATAATCAATTGAATGTTTCAGACAGAGAATGGTCT CCAAGGGAGAGG
P182	ACTGATCCTCCGGCCGACGAGACACTATTTGATGCCGCTTTGCCACTA CCTGATCCCTTG

*RNA oligonucleotides have U in their sequence and DNA oligonucleotides have T in their sequence.

Supplementary Table 5. Reagents used for Cas9-mediated genome editing.

Allele name	CRISPR edit	DNA template for sgRNA transcription or crRNA sequence	Homology repair dsDNA or ssDNA template	Length of homology repair template	Concentration in pmol/µl of 1st & 2nd sgRNA or crRNA; homology repair template; *dpy-10* sgRNA# or crRNA; *dpy-10* homology repair template
+	*dpy-10(-)* in wild type	P57 (FOR), P43 (REV)	P58 (ssDNA)	100 b	-; -; -; 3.05; 0.66
T	*dpy-10(-)* in *oxSi487*	P57 (FOR), P43 (REV)	P58 (ssDNA)	100 b	-; -; -; 3.05; 0.66
T *	*mCherry* mutation in *oxSi487*$	P64 (FOR), P43 (REV), P163 (FOR)	Left: P65 + P66, Right: P67 + P68, Fusion: P69 + P70	309 bp	1.6; 1.4; 0.12; 1.3; 0.66
*TΔ**	*mCherry* mutation in *jamSi19* (*TΔ*)	P46 (FOR), P43 (REV)	P50 (ssDNA)	60 b	6.05; -; 8.85; 3.05; -
*TΔΔ**	*mCherry* mutation in *jamSi25* (*TΔΔ*)	P46 (FOR), P43 (REV)	P50 (ssDNA)	60 b	6.05; -; 8.85; 3.05; -
TΔ	Deletion of *gfp* and *tbb-2* 3’	P59 (FOR), P43 (REV)	Left: P60 + P61,	1074 bp	2.96; -; 0.08; 3.05; 0.66

	utr from *oxSi487*		Right: P62 + P52, Fusion: P63 + P54		
TΔΔ	Deletion of *Punc-119* from *jamSi19* (*TΔ*)	P55 (FOR), P43 (REV)	P56 (ssDNA)	60 b	8.4; -; 1.53; 8.16; 1.52
TΔΔΔ	Deletion of *h2b* from *jamSi25* (*TΔΔ*)	P42 (FOR), P43 (REV)	Left: P44 + P45, Right: P47 + P48, Fusion: P80 + P81	1604 bp	11.16; 12.87; 0.31; 2.89; 0.62
TcherryΔpi N	Deletion of *mCherry* C-terminus (*TcherryΔpi*)	P164 (crRNA), P165 (crRNA)	P168 (ssDNA)	70 b	4.0; 4.0; 24; 2.4; 100
TcherryΔpi C	Deletion of *mCherry* N-terminus (*TcherryΔpi*)	P166 (crRNA), P167 (crRNA)	P169 (ssDNA)	70 b	18.6; 11.2; 24; 2.4; 100
TcherryΔpi exon4	Deletion of three *mCherry* exons from *jamSi39* (*TcherryΔpi*)	P173 (crRNA), P174 (crRNA)	P175 (ssDNA)	70 b	4.9; 4.9; 3.6; 2.4; 100
TΔorf	Deletion of *mCherry* ORF from *jamSi37* (*Tcherry*)	P170 (crRNA), P171 (crRNA)	P172 (ssDNA)	70 b	4.8; 4.8; 25; 2.4; 100
iT	*dpy-2(-)* repair in *iT dpy-2(-)*	P42 (FOR), P43 (REV)	P101 (ssDNA)	60 b	7.2; -; 0.6; -; -
rde-8(-)	*rde-8* mutation in *iT*	P100 (FOR), P43 (REV), P102 (FOR)	P103 (ssDNA)	60 b	8.1; 10.9; 13.5; 6.9; 6.5
set-32(-)	*set-32* mutation in *iT*	P104 (FOR), P43 (REV), P105 (FOR)	P106 (ssDNA)	60 b	3.9; 3.9; 7.5; 2.8; 7.5
heri-1(-)	*heri-1* mutation in *iT*	P107 (FOR), P43 (REV), P108 (FOR)	P109 (ssDNA)	60 b	3.7; 3.7; 7.5; 2.3 crRNA (P110), 2.7 tracrRNA (P111); 7.5
mcherry::mex-5	*mcherry* N-terminal fusion	P180 (crRNA)	P181 (FOR) P182 (REV)	958 b	4.2; -; 0.35; 2.7; 6.5

[$]refers to cases where the resulting edit was not the originally intended edit and therefore does not relate to the reagents injected.
[#]*dpy-10* sgRNA was transcribed in vitro using a DNA template generated using primers P57 (forward) and P43 (reverse).

Supplementary References

1. Hollick, J. B. Paramutation and related phenomena in diverse species. *Nat. Rev. Genet* **18**, 5–23 (2017).

2. de Vanssay, A., Bougé, A. L., Boivin, A., Hermant, C., Teysset, L., Delmarre, V., Antoniewski, C., & Ronsseray, S. Paramutation in Drosophila linked to emergence of a piRNA-producing locus. *Nature* **490**, 112–115 (2012).

3. Brink, R. A. A Genetic Change Associated with the *R* Locus in Maize Which Is Directed and Potentially Reversible. *Genetics* **41** 872–889 (1956).

4. Chandler, V. L., Eggleston, W. B., and Dorweiler, J. E. Paramutation in maize. *Plant Mol. Biol* **43**, 121-145 (2000).

5. Rassoulzadegan, M., Grandjean, V., Gounon, P., Vincent, S., Gillot, I., and Cuzin, F. RNA-mediated non-mendelian inheritance of an epigenetic change in the mouse. *Nature* **441**, 469–474 (2006).

6. Kidwell, M. G., Kidwell, J. F., and Sved, J. A. Hybrid Dysgenesis in *DROSOPHILA MELANOGASTER*: A Syndrome of Aberrant Traits Including Mutation, Sterility and Male Recombination. *Genetics* **86**, 813–833 (1977).

7. Kermicle, J. L., Eggleston, W. B., and Alleman, M. Organization of paramutagenicity in R-stippled maize. *Genetics* **141**, 361–372 (1995).

8. Stam, M., Belele, C., Dorweiler, J. E., and Chandler, V. L. Differential chromatin structure within a tandem array 100 kb upstream of the maize *b1* locus is associated with paramutation. *Genes Dev* **16**, 1906–1918 (2002).

9. Belele, C. L., Sidorenko, L., Stam, M., Bader, R., Arteaga-Vazquez, M. A., and Chandler, V. L. Specific tandem repeats are sufficient for paramutation-induced trans-generational silencing. *PLoS Genet* **9**, e1003773 (2013).

10. Diag, A., Schilling, M., Klironomos, F., Ayoub, S., & Rajewsky, N. Spatiotemporal m(i)RNA architecture and 3' UTR regulation in the C. elegans germline. *Dev Cell* **47**, 785-800 (2018).

11. Wedeles, C. J., Wu, M. Z., and Claycomb, J. M. Protection of germline gene expression by the *C. elegans* argonaute CSR-1. *Dev. Cell* **27**, 664–671 (2013).

12. Ashe, A., Sapetschnig, A., Weick, E. M., Mitchell, J., Bagijn, M. P., Cording, A. C., Doebley, A. L., Goldstein, L. D., Lehrbach, N. J., Le Pen, J., et al. piRNAs can trigger a multigenerational epigenetic memory in the germline of *C. elegans*. *Cell* **150**, 88–99 (2012).

13. Lee, H. C., Gu, W., Shirayama, M., Youngman, E., Conte Jr., D., Mello, C. C. *C. elegans* piRNAs mediate the genome-wide surveillance of germline transcripts. *Cell* **150**, 78–87 (2012).

14. Luteijn, M. J., van Bergeijk, P., Kaaij, L. J., Almeida, M. V., Roovers, E. F., Berezikov, E., and Ketting, R. F. Extremely stable Piwi-induced gene silencing in *Caenorhabditis elegans*. *EMBO J.* **31**, 3422–3430 (2012).

15. Ishidate, T., Ozturk, A. R., Durning, D. J., Sharma, R., Shen, E. Z., Chen, H., Seth, M., Shirayama, M., and Mello, C. C. ZNFX-1 functions within perinuclear nuage to balance epigenetic signals. *Mol. Cell* **70**, 639–649 (2018).

16. Shirayama, M., Seth, M., Lee, H. C., Gu, W., Ishidate, T., Conte Jr., D., and Mello, C. C. piRNAs initiate an epigenetic memory of nonself RNA in the *C. elegans* germline. *Cell* **150**, 65–77 (2012).

17. Seth, M., Shirayama, M., Gu, W. T., Ishidate, T., Conte Jr., D., and Mello, C. C. The *C. elegans* CSR-1 argonaute pathway counteracts epigenetic silencing to promote germline gene expression. *Dev. Cell* **27**, 656–663 (2013).

18. Seth, M., Shirayama, M., Tang, W., Shen, E. Z., Tu, S., Lee, H. C., Weng, Z., and Mello, C. C. The coding regions of germline mRNAs confer sensitivity to Argonaute regulation in *C. elegans*. *Cell Rep* **27**, 2254-2264 (2018).

19. Bagijn, M. P., Goldstein, L. D., Sapetschnig, A., Weick, E. M., Bouasker, S., Lehrbach, N. J., Simard, M. J., and Miska, E. A. Function, targets, and evolution of *Caenorhabditis elegans* piRNAs. *Science* **337**, 574–578 (2012).

20. Buckley, B. A., Burkhart, K. B., Gu, S. G., Spracklin, G., Kershner, A., Fritz, H., Kimble, J., Fire, A., and Kennedy, S. A nuclear Argonaute promotes multigenerational epigenetic inheritance and germline immortality. *Nature* **489**, 447-451 (2012).

21. Ni, J. Z., Kalinava, N., Chen, E., Huang, A., Trinh, T., and Gu, S. G. A transgenerational role of the germline nuclear RNAi pathway in repressing heat stress-induced transcriptional activation in *C. elegans*. *Epigenetics Chromatin* **9**, 3 (2016).

22. Batista, P. J, Ruby, J. G., Claycomb, J. M., Chiang, R., Fahlgren, N., Kasschau, K. D., Chaves, D. A., Gu, W., Vasale, J. J., Duan, S., et al. PRG-1 and 21U-RNAs interact to form the piRNA complex required for fertility in *C. elegans*. *Mol. Cell* 31, 67–78 (2008).

23. de Albuquerque, B. F., Placentino, M., and Ketting, R. F. Maternal piRNAs are essential for germline development following de novo establishment of endo-siRNAs in *Caenorhabditis elegans*. *Dev. Cell* **34**, 448–456 (2015).

24. Phillips, C. M., Brown, K. C., Montgomery, B. E., Ruvkun, G., & Montgomery, T. A. piRNAs and piRNA-Dependent siRNAs Protect Conserved and Essential C. elegans Genes from Misrouting into the RNAi Pathway. *Dev. Cell* **34**, 457–465 (2015).

25. Frøkjær-Jensen, C., Jain, N., Hansen, L., Davis, M. W., Li, Y., Zhao, D., Rebora, K., Millet, J.R.M., Liu, X., Kim, S. K., Dupuy, D., Jorgensen, E. M., and Fire, A. Z. An abundant class of

non-coding DNA can prevent stochastic gene silencing in the *C. elegans* germline. *Cell* **166**, 343–357 (2016).

26. Johnson, C. L., and Spence, A. M. Epigenetic licensing of germline gene expression by maternal RNA in *C. elegans*. *Science* **333**, 1311–1314 (2011).

27. Leopold, L. E., Heestand, B. N., Seong, S., Shtessel, L., Ahmed, S. Lack of pairing during meiosis triggers multigenerational transgene silencing in *Caenorhabditis elegans*. *Proc. Natl. Acad. Sci. USA* **112**, E2667–E2676 (2015).

28. Shiu, P. K., Raju, N. B., Zickler, D., and Metzenberg, R. L. Meiotic silencing by unpaired DNA. *Cell* **107**, 905-916 (2001).

29. Schedl, T., and Kimble, J. *fog-2*, a germ-line-specific sex determination gene required for hermaphrodite spermatogenesis in *Caenorhabditis elegans*. *Genetics* **119**, 43-61 (1988).

30. Marré, J., Traver, E. C., Jose and A. M. Extracellular RNA is transported from one generation to the next in *Caenorhabditis elegans*. *Proc. Natl. Acad. Sci. USA* **113**, 12496–12501 (2016).

31. Wang, E., and Hunter, C. P. SID-1 functions in multiple roles to support parental RNAi in *Caenorhabditis elegans*. *Genetics* **207**, 547–557 (2017).

32. Almeida, M. V., Andrade-Navarro, M. A., and Ketting, R. F. Function and evolution of nematode RNAi pathways. *Noncoding RNA* **5**, 8 (2019).

33. Shukla, A., Yan, J., Pagano, D. J., Dodson, A. E., Fei, Y., Gorham, J., Seidman, J. G., Wickens, M., and Kennedy, S. poly(UG)-tailed RNAs in genome protection and epigenetic inheritance. *Nature* **582**, 283–288 (2020).

34. Phillips, C. M., Montgomery, T. A., Breen, P. C., and Ruvkun, G. MUT-16 promotes formation of perinuclear mutator foci required for RNA silencing in the *C. elegans* germline. *Genes Dev.* **26**, 1433–1444 (2012).

35. Almeida, M. V., de Jesus Domingues, A. M., and Ketting, R. F. Maternal and zygotic gene regulatory effects of endogenous RNAi pathways. *PLoS Genet* **15**, e1007784 (2019).

36. Reed, K. J., Svendsen, J. M., Brown, K. C., Montgomery, B. E., Marks, T. N., Vijayasarathy, T., Parker, D. M., Nishimura, E. O., Updike, D. L., and Montgomery, T. A. Widespread roles for piRNAs and WAGO-class siRNAs in shaping the germline transcriptome of Caenorhabditis elegans. *Nucleic Acids Res* **48**, 1811-1827 (2020).

37. Zhang, D., Tu, S., Stubna, M., Wu, W. S., Huang, W. C., Weng, Z., and Lee, H. C. The piRNA targeting rules and the resistance to piRNA silencing in endogenous genes. *Science* **359**, 587-592 (2018).

38. Sha, K. and Fire, A. Imprinting capacity of gamete lineages in Caenorhabditis elegans. *Genetics* **170**, 1633–1652 (2005).

39. Hadchouel, M., Farza, H., Simon, D., Tiollais, P., and Pourcel, C. Maternal inhibition of hepatitis B surface antigen gene expression in transgenic mice correlates with de novo methylation. *Nature* **329**, 454–456 (1987).

40. Dodson, A. E., and Kennedy, S. Germ granules coordinate RNA-Based epigenetic inheritance pathways. *Dev. Cell* **50**, 704-715 (2019).

41. van der Graaf, A., Wardenaar, R., Neumann, D. A., Taudt, A., Shaw, R. G., Jansen, R. C., Schmitz, R. J., Colomé-Tatché, M., and Johannes, F. Rate, spectrum, and evolutionary dynamics of spontaneous epimutations. *Proc. Natl. Acad. Sci. USA* **112**, 6676-6681 (2015).

42. Spracklin, G., Fields, B., Wan, G., Becker, D., Wallig, A., Shukla, A., and Kennedy, S. The RNAi inheritance machinery of *Caenorhabditis elegans*. *Genetics* **206**, 1403-1416 (2017).

43. Fire, A., Xu, S., Montgomery, M. K., Kostas, S. A., Driver, S. E., and Mello, C. C. Potent and specific genetic interference by double-stranded RNA in Caenorhabditis elegans. *Nature* **391**, 806–811 (1998).

44. Burton, N.O., Burkhart, K.B., and Kennedy, S. Nuclear RNAi maintains heritable gene silencing in Caenorhabditis elegans. *Proc Natl Acad Sci USA* **108**, 19683–19688 (2011).

45. Burkhart, K.B., Guang, S., Buckley, B.A., Wong, L., Bochner, A.F., Kennedy S. A. pre-mRNA-associating factor links endogenous siRNAs to chromatin regulation. *PLoS Genet* **7**, e1002249 (2011).

46. Guang, S., Bochner, A.F., Burkhart, K.B., Burton, N., Pavelec, D.M., and Kennedy, S. Small regulatory RNAs inhibit RNA polymerase II during the elongation phase of transcription. *Nature* **465**, 1097–1101 (2010).

47. Tabara, H., Sarkissian, M., Kelly, W.G., Fleenor, J., Grishok, A., Timmons, L., Fire, A., and Mello, C.C. The *rde-1* gene, RNA interference, and transposon silencing in *C. elegans*. *Cell* **99**, 123–132 (1999).

48. Winston, W.M., Molodowitch, C., and Hunter, C.P. Systemic RNAi in C. elegans requires the putative transmembrane protein SID-1. *Science* **295**, 2456–2459 (2002).

49. Mao, H., Zhu, C., Zong, D., Weng, C., Yang, X., Huang, H., Liu, D., Feng, X., and Guang, S. The Nrde Pathway Mediates Small-RNA-Directed Histone H3 Lysine 27 Trimethylation in Caenorhabditis elegans. *Curr. Biol* **25**, 2398–2403 (2015).

50. Wan, G., Fields, B.D., Spracklin, G., Shukla, A., Phillips, C.M., and Kennedy, S. Spatiotemporal regulation of liquid-like condensates in epigenetic inheritance. *Nature* **557**, 679–683 (2018).

51. Xu, F., Feng, X., Chen, X., Weng, C., Yan, Q., Xu, T., Hong, M., and Guang, S. A Cytoplasmic Argonaute Protein Promotes the Inheritance of RNAi. *Cell Rep* **23**, 2482–2494 (2018).

52. Timmons, L., Tabara, H., Mello, C.C., and Fire, A.Z. Inducible systemic RNA silencing in *Caenorhabditis elegans*. *Mol. Biol. Cell* **14**, 2972–83 (2003).

53. Sapetschnig, A., Sarkies, P., Lehrbach, N. J., and Miska, E. A. Tertiary siRNAs mediate paramutation in *C. elegans*. *PLoS Genet* **11**, e1005078 (2015).

54. Ashe, A., Sarkies, P., Le Pen, J., Tanguy, M., and Miska, E.A. Antiviral RNA Interference against Orsay Virus Is neither Systemic nor Transgenerational in *Caenorhabditis elegans*. *J Virol* **89**, 12035–12046 (2015).

55. Grishok, A., Tabara, H., and Mello, C.C. Genetic requirements for inheritance of RNAi in *C. elegans*. *Science* **287**, 2494–2497 (2000).

56. Perales, R., Pagano, D., Wan, G., Fields, B. D., Saltzman, A. L., and Kennedy, S.G. Transgenerational epigenetic inheritance is negatively regulated by the HERI-1 chromodomain protein. *Genetics* **210**, 1287–1299 (2018).

57. Lev, I., Seroussi, U., Gingold, H., Bril, R., Anava, S., and Rechavi, O. MET-2-dependent H3K9 methylation suppresses transgenerational small RNA inheritance. *Curr. Biol* **27**, 1138–1147 (2017).

58. Houri-Ze'evi, L., Korem, Y., Sheftel, H., Faigenbloom, L., Toker, I.A., Dagan, Y., Awad, L., Degani, L., Alon, U., and Rechavi, O. A tunable mechanism determines the duration of the transgenerational small RNA inheritance in *C. elegans*. *Cell* **165**, 88-99 (2016).

59. Weiser, N.E., Yang, D.X., Feng, S., Kalinava, N., Brown, K.C., Khanikar, J., Freeberg, M.A., Snyder, M.J., Csankovszki, G., Chan, R.C., et al. MORC-1 integrates nuclear RNAi and transgenerational chromatin architecture to promote germline immortality. *Dev. Cell* **22**, 408–423 (2017).

60. Woodhouse, R.M., Buchmann, G., Hoe, M., Harney, D.J., Low, J.K.K., Larance, M., Boag, P.R., and Ashe, A. Chromatin modifiers SET-25 and SET-32 are required for establishment but not long-term maintenance of transgenerational epigenetic inheritance. *Cell Rep* **20**, 2259–2272 (2018).

61. Vastenhouw, N.L., Brunschwig, K., Okihara, K.L., Müller, F., Tijsterman, M., and Plasterk, R.H. Gene expression: long-term gene silencing by RNAi. *Nature* **442**, 882 (2006).

62. Lev, I., Gingold, H., and Rechavi, O. H3K9me3 is required for inheritance of small RNAs that target a unique subset of newly evolved genes. *eLife* **14**, e40448 (2019).

63. Devanapally, S., Ravikumar, S., Jose, A.M. Double-stranded RNA made in *C. elegans* neurons can enter the germline and cause transgenerational gene silencing. *Proc. Natl. Acad. Sci. USA* **112**, 2133–2138 (2015).

64. Conine, C. C., Moresco, J. J., Gu, W., Shirayama, M., Conte Jr., D., Yates, J. R., and Mello, C.C. Argonautes promote male fertility and provide a paternal memory of germline gene expression in *C. elegans*. *Cell* **155**, 1532-1544 (2013).

65. Bennett, S. T., Wilson, A. J., Esposito, L., Bouzekri, N., Undlien, D. E., Cucca, F., Nisticò, L., Buzzetti, R., Bosi, E., Pociot, F. et al. Insulin VNTR allele-specific effect in type 1 diabetes depends on identity of untransmitted paternal allele. The IMDIAB Group. *Nat. Genet* **17**, 350–352 (1997).

66. Brennecke, J., Malone, C. D., Aravin, A. A., Sachidanandam, R., Stark, A., and Hannon, G. J. An epigenetic role for maternally inherited piRNAs in transposon silencing. *Science* **322**, 1387–1392 (2008).

67. Duncan, I. W. Transvection effects in *Drosophila*. *Annu. Rev. Genet* **36**, 521–556 (2002).
