# Supplementary material for: Mating can initiate stable RNA silencing that overcomes epigenetic recovery
Source: Nat Commun. 2021 Jul 9;12:4239. doi: 10.1038/s41467-021-24053-4 (PMC8270896; doi:10.1038/s41467-021-24053-4)
Supplement: Supplementary file 4 — Supplementary Data 2 [file 41467_2021_24053_MOESM4_ESM.pdf]

Sat Mar 13, 2021 0:48 EST
gfpT.ape from 1 to 711
Alignment to
gtbp_mCherry_var.ape from 1 to 705

Matches(|):426
Mismatches(#):216
Gaps( ):132
Unattempted(.):0

```
  1 AGT---AAAGG----AGAAGA--ACTTTTCACT------GGAGTTGTCCCAATTCTTGTTGAAT-TAGATGGTGATGTTAATGGGCACAAATTTTCTGTC 84
  1 ~GTCTCAAAGGGTGAAGAAGATAACATGGCAATTATTAAAGAGTTTATGCGTTTCAAGGTGCATATGGAGGGATCTGTCAATGGGCATGAGTT---TGAA 96

 85 AGTGGAGAGGGTGAAGGTG-ATGC--AACAT---ACGGAAAACTTACCCTTAAATTTATTTGCACTA---CTGGAAAACTACCTGTTC-CATGGCCAACA 174
 97 ATT---GAAGGTGAAGGAGAAGGCCGACCATATGAGGGAACACAAACCGCAAAACTAAAGGTAACTAAAGGCGGACCATTACC-ATTCGCCTGG--GACA 190

175 -CTTGTCACTAC-TTTC-TGTTATGGTGTTCAATGCTTCT-CGAGATACCCAGATCATATGAAACGGCATGACTTTTTCAAGAGTGCCATGCCCGAAGG- 269
191 TCCTCTCTCCACAGTTCATG-TATGGAAGT-AAAGCTTATGTTAAACATCCGG--CAGAT-ATAC--CA-GATTATTTGAA-ACTTTCATTCCCGGAGGG 281

270 --TTATGTACAGGAAAGAACTAT-ATTTTTCAAAGATGACGG-GAACTACAAGACACGTG-CTGAAG--TCAAG-TTTGAAGGT-GATACCCTTGTTAAT 360
282 TTTTAAGT--GGGAACGCGTAATGAATTTT----GAAGACGGAGGAGT-TGTTACA-GTGACGCAAGACTCCAGCCTCCAAGATGGAGA-ATTTATTTAT 372

361 AGAATCGAGTTAAAAGGTATTGATTTTAAAGAAGATGGAAACATTCT--TGGACACAAAT-TGGAAT-ACAA-CTATAACTCACACAATGTATAC--ATC 453
373 AAAGTCAAACTTCGAGGAACGAATTTCCCCTCGGATGGACCTGTTATGCAGAAGAAGACTATGGGATGGGAAGCTTCAAGTGAAAGAATGTACCCTGAAG 472

454 ATG--GCAGACAAACAAAAGAATGGAATCAAAG--TTAACTTCAAAATTAGACACAACATTGAAGATGGAAGCGTTCAACTAGCAGACCA-TTATCAA-- 546
473 ACGGTGCTCTTAAGGGAGAGATT--AAACAACGTCTTAAATTGAAAGATGG--AGGACATT-ACGAT----GC--TGAGGT-GAAGACAACTTA-CAAAG 559

547 -CAAAATACTCCAATTGGC-GATGGCCCTGTCCTTTTACCAGACAACCATTACCTGTCCACA-CAATCTGCCCTTTCGAAAGATCCCAACGAAAAGAGAG 643
560 CCAAAAAA--CCAGTT--CAGCTG--CCAG------GAGCGTACAA--------TGTTAATATTAAACTG-GATATC---ACCTCCC-AC--AACGAG-G 631

644 ACCACATGGTCCTTCTTGAGTTTGTAACAGCTGCTGGG----ATT--ACACATGGCATGGATGAACTATACAAA 711
632 ATTACACTATCGTTGAGCAATATGAAAGAGCTGAAGGGCGGCACTCGACAGGTGGCATGGATGAATTGTATAAG 705
```
