# Supplementary material for: Mating can initiate stable RNA silencing that overcomes epigenetic recovery
Source: Nat Commun. 2021 Jul 9;12:4239. doi: 10.1038/s41467-021-24053-4 (PMC8270896; doi:10.1038/s41467-021-24053-4)
Supplement: Supplementary file 5 — Supplementary Data 3 [file 41467_2021_24053_MOESM5_ESM.pdf]

```
Sat Mar 13, 2021  0:50 EST
gfpT.ape from 1 to 711
Alignment to
gtbp_rfp.ape from 1 to 714

Matches(|):408
Mismatches(#):229
Gaps( ):151
Unattempted(.):0

  1 ~AG--TAAAGGAGAAGAACTTTTCACTGGAG---TTGTCCCA--ATTCTTGTTGAATTAGATGGTGATGTTAATGGGCACAAATT-TTCTGTCAGTGGAG 91
  1 GTGTCTAAGGGCGAAGAGCTGATTA-AGGAGAACATG-CACATGAAGC-TGT--ACATGGAGGGCACCGTGAACAACCACCACTTCAAATG-CACATCCG 94

 92 AGGGTGAAGGTGATGCAACATAC---GGAAAACTTACCCTTA-AATTTATTTGCACTA--CTGGAAAACTA-CCTGTTCCATGGCCAACACTTGTCACTA 184
 95 AGGGCGAAGG-CAAGC--CCTACGAGGGCACCCAGACCATGAGAATCAAGGTGGTCGAGGGCGGCCCTCTCCCCTTCGCCTTTGACATC-CTGGCTACCA 190

185 -CTTTCTGTTATGGTGTTCA-ATGCTTC-TCGA-GATACCCAGATCATATGAAACGGCATGACTTTTTCAAG-AGTGCCATGCCCGAAGGTTATGTACA- 278
191 GCTTCATG-TACGG-CAGCAGAACCTTCATCAACCACACCCAGGGCAT------C--CCCGACTTCTTTAAGCAGT-CCTTTCCTGAGGGCT-T-CACAT 277

279 -GGAAAGA---ACTATATTTTTCAAAGATGACGGGAAC-TACAAGACACG-TGCTGAAGTCA--AGTTTGAAGGTGATACCCTTGT-TAA--TAGAA--T 365
278 GGGAGAGAGTCACCACAT------ACGAAGACGGGGGCGTGC-TGAC-CGCTACCCAGGACACCAGCCTCCAGG----ACGGCTGTCTCATCTACAACGT 365

366 CGAGTTAAAA--GGT-A--TT--GAT----------TTTAAAGAAGATGGAAACATT---CTTGGACACAAATTGGAATACAACTATAACTCACACAATG 445
366 CAAGATCAGAGGGGTGAACTTCCCATCCAACGGCCCTGTGATGCAGAAGAAAACACTCGGCTGGGAGGCCAATACCGAGA-TGCTGTACCCCGC----TG 460

446 TATACATCATGGCAGACAAACAAAAGA-ATGGAATCAAAGTTAACTTCAAAATTAGACACAACATTGAAGATGGAAGCGTTCAACTAGCAGA-CCATTAT 543
461 ACGGCGGCCTGGAAGGC--AGAAGCGACATGGCCCTGAAGCT--CGTGGGCGGGGGCCAC--C--TG-ATCTGCAA--TTTCAA-GACCACATACA-GAT 547

544 CAACAAAATACTCCAATTGGCGATGGCCCTGTCCTTTTACCAGACAACCATTACCTGTCCA-CACAATCTGCCCTTTCGAAAGATCCCAA-----CGAAA 637
548 C--CAAGAAAC-CC-----GCTAAGAACC--TCAAGATGCCCGGCGTCTACTA--TGTGGACCACAGACTG-------GAAAGAATCAAAGAGGCCGACA 628

638 AGAGAGACC-ACATGGTCCTTCTTGAGTT---TGT-AACAGCTGCTGGGATTACACATGGCA---TGG---ATGAAC-TA--TACAAA 711
629 A-AGAGACCTACGTCGAGCAGCACGAGGTGGCTGTGGCCAGATACTGCGACCTC-CCTAGCAAACTGGGGCACAAACTTAATTACGTA 714
```
