# Supplementary material for: Mating can initiate stable RNA silencing that overcomes epigenetic recovery
Source: Nat Commun. 2021 Jul 9;12:4239. doi: 10.1038/s41467-021-24053-4 (PMC8270896; doi:10.1038/s41467-021-24053-4)
Supplement: Supplementary file 6 — Supplementary Data 4 [file 41467_2021_24053_MOESM6_ESM.pdf]

```
Sat Mar 13, 2021  0:52 EST
Tcherry.ape from 1 to 720
Alignment to
gtbp_mCherry_var.ape from 1 to 705

Matches(|):557
Mismatches(#):148
Gaps( ):15
Unattempted(.):0

             *         *         *         *         *         *         *         *         *         *
  1 GTCTCCAAGGGAGAGGAGGATAACATGGCTATCATCAAGGAGTTCATGAGATTCAAGGTCCACATGGAGGGATCCGTCAACGGACACGAGTTCGAGATCG 100
    |||||#|||||#||#||#|||||||||||#||#||#||#|||||#|||#|#||||||||#||#|||||||||||#|||||#||#||#|||||#||#||#|
  1 GTCTCAAAGGGTGAAGAAGATAACATGGCAATTATTAAAGAGTTTATGCGTTTCAAGGTGCATATGGAGGGATCTGTCAATGGGCATGAGTTTGAAATTG 100
             *         *         *         *         *         *         *         *         *         *

             *         *         *         *         *         *         *         *         *         *
101 AGGGAGAGGGAGAGGGAAGACCATACGAGGGAACCCAGACCGCTAAGCTTAAGGTCACCAAGGGAGGACCACTTCCATTCGCTTGGGATATCCTTTCCCC 200
    |#||#||#|||||#||##|||||||#||||||||#||#|||||#||#||#|||||#||#||#||#|||||||#|#||||||||#|||||#|||||#||#||
101 AAGGTGAAGGAGAAGGCCGACCATATGAGGGAACACAAACCGCAAAACTAAAGGTAACTAAAGGCGGACCATTACCATTCGCCTGGGACATCCTCTCTCC 200
             *         *         *         *         *         *         *         *         *         *

             *         *         *         *         *         *         *         *         *         *
201 ACAGTTCATGTACGGATCCAAGGCTTACGTCAAGCACCCAGCTGATATCCCAGATTACCTTAAGCTTTCCTTCCCAGAGGGATTCAAGTGGGAGAGAGTC 300
    ||||||||||||#|||###||#|||||#||#||#||#||#||#|||||#||||||||##|#||#|||||#|||||#|||||#||#|||||||||##|#||#
201 ACAGTTCATGTATGGAAGTAAAGCTTATGTTAAACATCCGGCAGATATACCAGATTATTTGAAACTTTCATTCCCGGAGGGTTTTAAGTGGGAACGCGTA 300
             *         *         *         *         *         *         *         *         *         *

             *         *         *         *         *         *         *         *         *         *
301 ATGAACTTCGAGGATGGAGGAGTCGTCACCGTCACCCAGGATTCCTCCCTTCAGGATGGAGAGTTCATCTACAAGGTCAAGCTTAGAGGAACCAACTTCC 400
    |||||#||#||#||#||||||||#||#||#||#||#||#||#|||##|||#||#||||||||#||#||#||#||#|||||#|||#|||||||#||#||||
301 ATGAATTTTGAAGACGGAGGAGTTGTTACAGTGACGCAAGACTCCAGCCTCCAAGATGGAGAATTTATTTATAAAGTCAAACTTCGAGGAACGAATTTCC 400
             *         *         *         *         *         *         *         *         *         *

             *         *         *         *         *         *         *         *         *         *
401 CATCCGATGGACCAGTCATGCAGAAGAAGACCATGGGATGGGAGGCTTCCTCCGAGAGAATGTACCCAGAGGATGGAGCTCTTAAGGGAGAGATCAAGCA 500
    |#||#||||||||#||#||||||||||||||#|||||||||||#|||||####||#|||||||||||#||#||#||#||||||||||||||||||#||#||
401 CCTCGGATGGACCTGTTATGCAGAAGAAGACTATGGGATGGGAAGCTTCAAGTGAAAGAATGTACCCTGAAGACGGTGCTCTTAAGGGAGAGATTAAACA 500
             *         *         *         *         *         *         *         *         *         *

             *         *         *         *         *         *         *         *         *         *
501 GAGACTTAAGCTTAAGGATGGAGGACACTACGATGCTGAGGTCAAGACCACCTACAAGGCTAAGAAGCCAGTCCAGCTTCCAGGAGCTTACAACGTCAAC 600
    ##|#|||||##|#||#|||||||||||#||||||||||||||#|||||#||#|||||#||#||#||#|||||#|||||#|||||||#|||||#||#||#
501 ACGTCTTAAATTGAAAGATGGAGGACATTACGATGCTGAGGTGAAGACAACTTACAAAGCCAAAAAACCAGTTCAGCTGCCAGGAGCGTACAATGTTAAT 600
             *         *         *         *         *         *         *         *         *         *

             *         *         *         *         *         *         *         *         *         *
601 ATCAAGCTTGATATCACCTCCCACAACGAGGATTACACCATCGTCGAGCAGTACGAGAGAGCTGAGGGAAGACACTCCACCGGAGGAATGGATGAGCTTT 700
    ||#||#||#|||||||||||||||||||||||||||||#|||||#|||||#||#||#||||||||#||##|#|||||#||#||#||#|||||||##|#|
601 ATTAAACTGGATATCACCTCCCACAACGAGGATTACACTATCGTTGAGCAATATGAAAGAGCTGAAGGGCGGCACTCGACAGGTGGCATGGATGAATTGT 700
             *         *         *         *         *         *         *         *         *         *

             *         *
701 ACAAGGGATCAGGTAGTGGC 720
    |#|||               
701 ATAAG~~~~~~~~~~~~~~~ 705
```
