# Supplementary material for: Mating can initiate stable RNA silencing that overcomes epigenetic recovery
Source: Nat Commun. 2021 Jul 9;12:4239. doi: 10.1038/s41467-021-24053-4 (PMC8270896; doi:10.1038/s41467-021-24053-4)
Supplement: Supplementary file 7 — Supplementary Data 5 [file 41467_2021_24053_MOESM7_ESM.pdf]

Sat Mar 13, 2021 0:53 EST
Tcherry.ape from 1 to 720
Alignment to
gtbp_rfp.ape from 1 to 714

Matches(|):489
Mismatches(#):184
Gaps( ):88
Unattempted(.):0

```
  1 GTCTCCAAGGGAGAGGAGGATAACATGGCTATCATCAAGGAGTTCATG-AGATTCAAGGTCCACATGGAGGGATCCGTCAACGGACACGAGTTC----G- 94
  1 GTGTCTAAGGGCGAAGAG-----C-TG------ATTAAGGAGAACATGCACA-TGAAGCTGTACATGGAGGGCACCGTGAACAACCACCACTTCAAATGC 87

 95 AGAT-CGAGGGAGAGGGAGAGGGAAGACCATACGAGGGAACCCAGACCGCTAAG-CTTAAGGTCACCAAGGGAGGACCACTTCCATTCGCTTGGGATATC 192
 88 ACATCCGAGGGCGA-----AGGCAAG-CCCTACGAGGGCACCCAGACC-ATGAGAATCAAGGTGGTCGAGGGCGGCCCTCTCCCCTTCGCCTTTGACATC 180

193 CTTTCCCCACAG-TTCATGTACGG-ATCCAAGGCTTACGTCAA--GCACCCAGCTGATATCCCAGATTACCTTAAGCTTTCCTTCCCAGAGGGATTCAAG 288
181 CTGGCTAC-CAGCTTCATGTACGGCAGCAGAACCTT-CATCAACCACACCCAG--GGCATCCCCGACTTCTTTAAGCAGTCCTTTCCTGAGGGCTTCACA 276

289 TGGGAGAGAGTCATGAACTTCGAGGATGGAGGAGTCGTCACCGTCACCCAGGATTCCTCCCTTCAGGATGGAGAGT-TCATCTACAAGGTCAAGCTTAGA 387
277 TGGGAGAGAGTCACCACATACGAAGACGGGGGCGTGCTGACCGCTACCCAGGACACCAGCCTCCAGGACGG-CTGTCTCATCTACAACGTCAAGATCAGA 375

388 GGAACCAACTTCCCATCCGATGGACCAGTCATGCAGAAGAAGACCATGGGATGGGAGGCTTCCTCCGAGAGAATGTACCCAGAGGATGGAGCTCT-TAAG 486
376 GGGGTGAACTTCCCATCCAACGGCCCTGTGATGCAGAAGAAAACACTCGGCTGGGAGGCCAATACCGAGATGCTGTACCCCGCTGACGGCGGCCTGGAAG 475

487 GGAGAGATCAAGCA-GAGACTTAAGCTTAAGGATGGAGGACA-CTACGATGCTG--AGGTCAAGACCACCTACAAGGCTAAGAAGCC-----AG----TC 573
476 GCAGA-AGCGA-CATGGCCCTGAAGCTCGTGGGCGGGGGCCACCT--GAT-CTGCAATTTCAAGACCACATACAGATCCAAGAAACCCGCTAAGAACCTC 570

574 CAGCTTCCAGGAGCTTACAACGTCAACATCAAGCTTGA---TATCA---CCTCCCACAACGAGGATTACACCATCGTCGAGCAGTACGAGAGAGCTGAGG 667
571 AAGATGCCCGGCGTCTACTATGTGGACCACAGACTGGAAAGAATCAAAGAGGCCGACAAAGAG------ACCTACGTCGAGCAGCACGAGGTGGCTGTGG 664

668 GAAGACACT----CCACCGGAGGAA--TGGATGAGCTTTACAA--GGGATCAGGTAGTGGC 720
665 CCAGATACTGCGACCTCCCTAGCAAACTGG--G-GC---ACAAACTTAATTACGTA~~~~~ 714
```
