# Supplementary material for: Mating can initiate stable RNA silencing that overcomes epigenetic recovery
Source: Nat Commun. 2021 Jul 9;12:4239. doi: 10.1038/s41467-021-24053-4 (PMC8270896; doi:10.1038/s41467-021-24053-4)
Supplement: Supplementary file 8 — Supplementary Data 6 [file 41467_2021_24053_MOESM8_ESM.pdf]

```
Sat Mar 13, 2021  0:55 EST
Tcherry.ape from 1 to 720
Alignment to
gtbp_gfp.ape from 1 to 711

Matches(|):429
Mismatches(#):214
Gaps( ):145
Unattempted(.):0

  1 ~GTCTCCAAGGGAGAGGAGGATAACA-TG--GCTATCATCAAGGAGTTCATGAGATTCAAGGTCCACATGGAGGGATCCGTCAACGGACACGAGTTCGAG 96
  1 AGT----AAAGGAGAAGAACTTTTCACTGGAGTTGTC-CCAA----TTCTTG---TTGAA--T-TAGATG--GTGAT--GTTAATGGGCACAAATTTTCT 81

 97 ATCGAG-GGAGAGGGAG-AGG-GAAG-ACCATACGAGGGAACCCAGACCGCTAAGCTTAAGGTCACCAAGGGAGGACCACTTCCATTCGCTTGGGATATC 192
 82 GTC-AGTGGAGAGGGTGAAGGTGATGCAACATAC---GGAAAACTTACCCTTAAATTTATTTGCACTACTGGAAAACTAC--CTGTTC-CATGGCCAACA 174

193 CTT-TCCCCACAGTTCATG-TACGG-ATCCAAGGCTTACGTCAAG-CACCCAGCTGATATCCCA-G--ATTACCTT---AAGCTTTCCTTCCCAGAGGGA 282
175 CTTGTCACTAC-TTTC-TGTTATGGTGTTCAATGCTT-C-TCGAGATACCCAGATCATATGAAACGGCATGACTTTTTCAAGAGTGCCATGCCCGAAGG- 269

283 TTCAAGT--GGGAGAGAGTCATGAACTTCGAGGATGGAGGAGTCGTCACCGTCAC--CCAGGATTCCTCCCTTCAGGATGGAGA-----GTTCATCTACA 373
270 TT-ATGTACAGGAAAGAACTATATTTTTCAAAGATGACGGGAACTACA-AGACACGTGCTGAAGT-CAAGTTTGAAGGT-GATACCCTTGTTAA--TAGA 363

374 AGGTCAAGCTTAGAGGAACCAACTTCCCATCCGATGG-ACCAGTCATG--CAGAA----GAAGAC----------CATGGGATGGAGGCTTCCTCCGAG 456
364 A--TCGAGTTAAAAGGTATTGATTTTAAAGAAGATGGAAACATTCTTGGACACAAATTGGAATACAACTATAACTCACACAAT-GTATACATCATGGCAG 460

457 AGAATGTACCCAGAGGATGGAGCTCTTAAGGGAGAGATCAAGCAGAGACTTAAGCTTAAGGATGGA---GGAC-ACTA-C-GATGCTGAGGTCAAGACCA 550
461 ACAA---A-CAAAAGAATGGA-ATC-AAAGTTA-ACTTCAAAATTAGACACAACATTGAAGATGGAAGCGTTCAACTAGCAGACCATTA--TCAACAAAA 551

551 -CCTACAA--GGCTAAGAAGCCAGTCCAGCTTCCAG-GAGC--TTACAAC-GTCAACATCAAGCTTG--ATATC---ACCTCCC-AC--AACGAG-GATT 634
552 TACTCCAATTGGCGATG-GCCCTGTCCTTTTACCAGACAACCATTAC--CTGTCCACA-CAATC-TGCCCTTTCGAAAGATCCCAACGAAAAGAGAGACC 646

635 ACACCAT-CGTCGAGCAGTACGAGAGAGCTG-AGGGAAGACACTCCACCGGAGGAATGGATGAGCTTTACAAGGGATCAGGTAGTGGC 720
647 ACATGGTCCTTCTTG-AGTTTGTAACAGCTGCTGGGATTACA---CA----TGGCATGGACGAACTATACAA---A~~~~~~~~~~~~ 711
```
