# Supplementary material for: Mating can initiate stable RNA silencing that overcomes epigenetic recovery
Source: Nat Commun. 2021 Jul 9;12:4239. doi: 10.1038/s41467-021-24053-4 (PMC8270896; doi:10.1038/s41467-021-24053-4)
Supplement: Supplementary file 9 — Description of Additional Supplementary Files [file 41467_2021_24053_MOESM9_ESM.docx]

Description of additional supplementary files

Title: Supplementary Data 1.

Description: Sequence alignment of gfp from T with gfp from gtbp-1::gfp

Title: Supplementary Data 2.

Description: Sequence alignment of gfp from T with mCherry variant from gtbp-1::mCherry-var

Title: Supplementary Data 3.

Description: Sequence alignment of gfp from T with rfp from gtbp-1::rfp

Title: Supplementary Data 4.

Description: Sequence alignment of mCherry from T with mCherry variant from gtbp-1::mCherry-var

Title: Supplementary Data 5.

Description: Sequence alignment of mCherry from T with rfp from gtbp-1::rfp

Title: Supplementary Data 6.

Description: Sequence alignment of mCherry from T with gfp from gtbp-1::gfp
