# Supplementary material for: Mating can initiate stable RNA silencing that overcomes epigenetic recovery
Source: Nat Commun. 2021 Jul 9;12:4239. doi: 10.1038/s41467-021-24053-4 (PMC8270896; doi:10.1038/s41467-021-24053-4)
Supplement: Supplementary file 10 — Reporting Summary [file 41467_2021_24053_MOESM10_ESM.pdf]

nature research

Corresponding author(s): Antony M Jose

Last updated by author(s): Apr 28, 2021

Reporting Summary

Nature Research wishes to improve the reproducibility of the work that we publish. This form provides structure for consistency and transparency in reporting. For further information on Nature Research policies, see our Editorial Policies and the Editorial Policy Checklist.

Statistics

For all statistical analyses, confirm that the following items are present in the figure legend, table legend, main text, or Methods section.

n/a	Confirmed	
☐	☒	The exact sample size (*n*) for each experimental group/condition, given as a discrete number and unit of measurement
☐	☒	A statement on whether measurements were taken from distinct samples or whether the same sample was measured repeatedly
☐	☒	The statistical test(s) used AND whether they are one- or two-sided<br>*Only common tests should be described solely by name; describe more complex techniques in the Methods section.*
☒	☐	A description of all covariates tested
☐	☒	A description of any assumptions or corrections, such as tests of normality and adjustment for multiple comparisons
☐	☒	A full description of the statistical parameters including central tendency (e.g. means) or other basic estimates (e.g. regression coefficient) AND variation (e.g. standard deviation) or associated estimates of uncertainty (e.g. confidence intervals)
☐	☒	For null hypothesis testing, the test statistic (e.g. *F*, *t*, *r*) with confidence intervals, effect sizes, degrees of freedom and *P* value noted<br>*Give P values as exact values whenever suitable.*
☒	☐	For Bayesian analysis, information on the choice of priors and Markov chain Monte Carlo settings
☒	☐	For hierarchical and complex designs, identification of the appropriate level for tests and full reporting of outcomes
☒	☐	Estimates of effect sizes (e.g. Cohen's *d*, Pearson's *r*), indicating how they were calculated

Our web collection on statistics for biologists contains articles on many of the points above.

Software and code

Policy information about availability of computer code

Data collection	NIS-Elements version 4.13 (Nikon) was used to acquire all wide-field images. Leica Application Suite Advance Fluorescence version 2.4.1 (LAS AF) was used to acquire confocal images.
Data analysis	Fiji version 2.0.0-rc-69/1.52p (NIH) and Photoshop version 21.2.4 (Adobe Creative Cloud) were used to adjust image clarity and quantify fluorescence - all samples were altered identically for comparison. Colocalization Colormap plugin within Fiji was used to measure smFISH signals. Matlab version R2019b (9.7.0), R version 3.5.3 and Microsoft Excel version 16.48 were used to plot fluorescence intensity (bar chart, rose plot, box and whisker plot, dot plot, line plot) and qPCR data. qPCR was performed using BIO-RAD CFX96 Real-Time System C1000 Thermal Cycler model number CFX96 Optics Module with a "Bio-Rad CFX Manager 3.1" software file version 3.1.15170823

For manuscripts utilizing custom algorithms or software that are central to the research but not yet described in published literature, software must be made available to editors and reviewers. We strongly encourage code deposition in a community repository (e.g. GitHub). See the Nature Research guidelines for submitting code & software for further information.

Data

Policy information about availability of data

All manuscripts must include a data availability statement. This statement should provide the following information, where applicable:

- Accession codes, unique identifiers, or web links for publicly available datasets
- A list of figures that have associated raw data
- A description of any restrictions on data availability

All data generated or analyzed during this study are included in this published article (and its supplementary information files). More than 10,000 images were generated during this study to document expression levels, which are available upon request. Source data are provided with this paper with corresponding figures noted.

Field-specific reporting

Please select the one below that is the best fit for your research. If you are not sure, read the appropriate sections before making your selection.

☒ Life sciences ☐ Behavioural & social sciences ☐ Ecological, evolutionary & environmental sciences

For a reference copy of the document with all sections, see nature.com/documents/nr-reporting-summary-flat.pdf

Life sciences study design

All studies must disclose on these points even when the disclosure is negative.

Sample size	Sample sizes chosen are comparable to or exceed other published studies that have similar work or use similar assays. Sample sizes varied based on availability of strains of particular genotype after crosses. Generally, more than 10 animals were analyzed for any experimental condition. In cases where more animals were available, a sample size in the range of ~100 was possible and used.
Data exclusions	No data were excluded from the analyses.
Replication	The replicates and trials for each experiment are as indicated in the manuscript. Unless indicated in the manuscript, experiments were performed once or the number of times indicated within source data (e.g., as independent cross plates for genetic crosses). Critical experiments were replicated by multiple authors (e.g., mating-induced silencing by 8 authors, pgl-1-dependent silencing by 2 authors). In these cases, results were reproducible.
Randomization	Within a given genotype, animals were selected at random. For pedigree analysis, the specific animals analyzed and their lineages are as indicated in the manuscript. Animals of a specific-stage (L4) were selected for mating experiments and passaging across generations was performed blindly unless noted in the manuscript.
Blinding	During image acquisition, blinding was not possible because the stark differences in fluorescence intensity between control and test animals was immediately obvious to the experimenter. As an alternative to blinding, all first and middle authors independently performed key experiments in multiple trials from the start of the experiment at the bench to the end of data collection. Data was quantified by identical methods for the same type of experiment. For ChIP-qPCR and RT-qPCR experiments, the experimenter was not blinded to the samples.

Reporting for specific materials, systems and methods

We require information from authors about some types of materials, experimental systems and methods used in many studies. Here, indicate whether each material, system or method listed is relevant to your study. If you are not sure if a list item applies to your research, read the appropriate section before selecting a response.

Materials & experimental systems

n/a	Involved in the study	
☐	☒	Antibodies
☒	☐	Eukaryotic cell lines
☒	☐	Palaeontology and archaeology
☐	☒	Animals and other organisms
☒	☐	Human research participants
☒	☐	Clinical data
☒	☐	Dual use research of concern

Methods

n/a	Involved in the study	
☒	☐	ChIP-seq
☒	☐	Flow cytometry
☒	☐	MRI-based neuroimaging

Antibodies

Antibodies used	anti-H3 antibody (Abcam, ab1791), anti-H3K9me1 antibody (Abcam, ab8896), anti-H3K9me2 antibody (Abcam, ab1220) or anti-H3K9me3 antibody (Abcam, ab8898). The amount of each antibody (in ug) added to 720 ul of the lysate is indicated in methods.
Validation	These antibodies were validated in Egelhofer, T. A., Minoda, A., Klugman, S., Lee, K., Kolasinska-Zwierz, P., Alekseyenko, A. A., Cheung, M. S., Day, D. S., Gadel, S., Gorchakov, A. A., et al. (2011). An assessment of histone-modification antibody quality. Nat. Struct. Mol. Biol. 18, 91-93.

Animals and other organisms

Policy information about studies involving animals; ARRIVE guidelines recommended for reporting animal research

Laboratory animals	Species- Caenorhabditis elegans, wild-type strain N2 Bristol background or mutant derivatives of N2, Sex - hermaphrodites, mutant females and males, Age - varied between experiments (L4-stage for mating; L4-staged and adults for imaging; L1, L2, L3, and L4 for smFISH).

Wild animals	The study did not involve wild animals.
Field-collected samples	The study did not involve samples collected from the field.
Ethics oversight	Recombinant DNA use was approved by the University of Maryland Office of Environmental Safety. No additional ethical approval was required because all work uses standard assays for the model nematode C. elegans.

Note that full information on the approval of the study protocol must also be provided in the manuscript.
